# Application of partial least squares discriminant analysis to mid-infrared spectral data for discriminating green and roasted coffee processed by different post-harvest methods: MATLAB- and R-based computational tools and modeling pipelines

**DOI:** 10.1016/j.dib.2026.113343

**Published:** 2026-10-14

**Authors:** Gentil A. Collazos-Escobar, Andrés F. Bahamón-Monje, Nelson Gutiérrez-Guzmán

**Affiliations:** a Centro Surcolombiano de Investigación en Café (CESURCAFÉ), Departamento de Ingeniería Agrícola, Universidad Surcolombiana, Neiva-Huila, 410001, Colombia; b University Institute of Food Engineering-FoodUPV, Universitat Politècnica de València, Camino de Vera s/n, València, 46022, Spain; c Departamento de Ingeniería Agroindustrial, Facultad de Ingeniería, Universidad Surcolombiana, Neiva, Huila, 410001, Colombia

**Keywords:** Mid-infrared spectroscopy, Non-invasive quality inspection, Full-spectrum and feature-extracted modeling, Multivariate statistical classification, Model-based decision making

## Abstract

This article presents a mid-infrared spectral dataset of green and roasted specialty coffee subjected to wet (W), semi-dry (SD), and dry (Dr) post-harvest processing, accompanied by MATLAB-based feature-extraction tools and R-based Partial Least Squares Discriminant Analysis (PLS-DA) modeling pipelines for discriminating coffee according to its post-harvest processing method. The dataset comprises raw, baseline-corrected, Standard Normal Variate (SNV), Multiplicative Scatter Correction (MSC), and Savitzky-Golay first- (D1) and second-derivative (D2) spectra, enabling the investigation of spectral variability associated with post-harvest processing and roasting, as well as the influence of spectral preprocessing on classification performance. The MATLAB routines extract six quantitative spectral features: integral (INT), energy (ENG), standard deviation (STD), skewness (SKE), kurtosis (KURT), and entropy (ENT). The R workflow provides an interactive framework for PLS-DA modeling based on four predictor representations: (i) full-spectrum data, (ii) full-spectrum data restricted to predefined spectral regions, (iii) features extracted from the entire spectrum, and (iv) features extracted from predefined spectral regions. Models can be developed using green coffee, roasted coffee, or both stages and configured for either multiclass discrimination among W, SD, and Dr processing methods or pairwise binary discrimination (W vs. Dr, W vs. SD, and SD vs. Dr). The computational pipeline integrates class definition, stratified training-validation partitioning, model calibration and prediction, K-fold out-of-fold cross-validation (OOF-CV), holdout validation, confusion matrices, overall and class-specific classification metrics, and visualization of scores, loadings, variable importance in projection (VIP), and model diagnostics. Metadata describing the post-harvest processing method and coffee stage are included to facilitate sample traceability and model interpretation. The integrated dataset and computational tools provide a reproducible framework for investigating the potential of mid-infrared spectroscopy to discriminate coffee post-harvest processing methods and support further developments in coffee authentication, quality control, and the application of chemometric methods to agri-food spectral data.

Specifications Table

**Table:** 

Subject	Food Science
Specific subject area	Food engineering, Food Science and Technology, Optimization in food engineering, Statistical analysis
Type of data	Excel files: (raw mid-infrared spectra, baseline-corrected spectra, SNV-corrected spectra, MSC-corrected spectra, first-D1 and second-D2 Savitzky-Golay derivative spectra). MATLAB scripts (Feature extraction, Feature extraction considering specific spectral region, Integral, Energy, Standard deviation, Skewness, Kurtosis and Entropy). R scripts (PLS-DA data-driven modeling routines). README (best practice guide of computer tools)
Data collection	Initial characterization (moisture gravimetrically obtained, water activity; obtained using a Vapor Sorption Analyzer, Color in CieLab color system using spectrocolorimeter). Mid-infrared spectra (obtained by Attenuated Total Reflectance-Fourier Transform Infrared; ATR-FTIR).
Data source location	All experimental data were obtained at the Centro Surcolombiano de Investigación en Café (CESURCAFÉ) of the Universidad Surcolombiana (Neiva, Huila, Colombia).
Data accessibility	Repository name: Mendeley Data Data identification number: 10.17632/9×3ns2f6vr.4 Direct URL to data: https://data.mendeley.com/datasets/9×3ns2f6vr/4
Related research article	Not applicable.

## 1 Value of the Data

• The dataset provides Fourier transform mid-infrared spectra of green and roasted specialty coffee samples processed by wet, semi-dry and dry post-harvest methods, enabling the characterization of spectral variability associated with both post-harvest processing and spectral-related transformations of green coffee during roasting.
• The availability of raw and preprocessed spectral data, including baseline-corrected, SNV, MSC, D1 and D2 allows researchers to directly assess the influence of spectral preprocessing on coffee classification performance of mathematical models.
• The dataset includes both full-spectrum information and feature-extracted variables derived from MATLAB-based routine. These approaches enable comparison between a modeling process carried out using the complete spectral fingerprinting and reduced feature-based extraction for chemometric modeling.
• The accompanying MATLAB scripts provide reusable tools for extracting quantitative spectral variables from different preprocessed spectral matrices, supporting reproducible feature extraction and facilitating the development of robust datasets for tuning classification models.
• The R-based modeling pipeline implements PLS-DA for discriminating specialty coffee according to post-harvest processing method. The script enables preprocessing selection, full-spectrum or feature-extracted modeling, green-only, roasted-only or combined-stage analysis, stratified training and validation partitioning, model calibration, prediction and performance assessment. These computational tools can also be extended to modeling tasks involving other agri-food products, supporting the development of data-driven quality monitoring systems.
• This dataset and its associated computational tools may be useful for food scientists, spectroscopists, chemometricians and coffee-sector stakeholders interested in coffee authentication, non-destructive quality control and the development of reproducible data-driven classification pipelines for agri-food spectral analysis.

## 2 Background

Coffee is one of the most widely consumed beverages worldwide and represents a major agricultural commodity with substantial economic, social and commercial relevance [1]. In specialty coffee production, quality is strongly influenced by harvesting and post-harvest processing practices, since wet, semi-dry and dry methods can induce differences in the chemical composition of coffee beans and, consequently, in their sensory profile, traceability and commercial value [2]. These differences may be further modified during roasting, where thermal reactions reshape the chemical matrix of the bean and generate additional compositional variability [3]. Therefore, analytical strategies capable of characterizing both green and roasted coffee are relevant for understanding the relationship between post-harvest processing, roasting-related transformations and final coffee quality.

Conventional coffee quality assessment commonly relies on manual inspection, sensory evaluation and targeted chemical analyses. Although these approaches remain important, they can be time-consuming, labor-intensive and difficult to implement as rapid screening tools for large sample sets. In this context, Fourier transform mid-infrared spectroscopy has emerged as a rapid, non-destructive and chemically informative technique for obtaining spectral fingerprints of food and agricultural matrices[[4], [5], [6], [7]]. Mid-infrared spectra provide information associated with functional groups and molecular vibrations related to carbohydrates, lipids, proteins, organic acids, phenolic compounds and other chemical constituents involved in coffee quality and processing differentiation [8,9].

The interpretation of spectral fingerprints requires appropriate computational strategies because infrared datasets are typically high-dimensional, collinear and affected by baseline shifts, scattering effects and signal variability [10]. Spectral preprocessing methods such as baseline correction, SNV, MSC and Savitzky-Golay derivatives are commonly applied to reduce non-informative variability and enhance chemically relevant spectral patterns [11]. In parallel, feature extraction strategies can summarize the spectral signal through quantitative variables related to intensity, energy, dispersion, asymmetry, peakedness and entropy, providing a reduced representation of the spectral profile that may complement full-spectrum modeling [12].

Chemometric classification methods, particularly PLS-DA, are well suited for modeling spectral datasets because they project high-dimensional predictor matrices into latent variables that maximize the relationship between spectral information and categorical responses [[13], [14], [15]]. This makes PLS-DA a suitable method for discriminating coffee samples according to post-harvest processing method while considering different spectral preprocessing strategies, data representations and coffee processing stages [16]. However, there is a lack in the literature regarding available datasets integrating green and roasted specialty coffee processed by different post-harvest processing methods, preprocessed mid-infrared spectra, feature-extracted variables and user-friendly pipeline based computational tools remain limited. In this work, a Fourier transform mid-infrared spectral dataset of green and roasted specialty coffee samples processed by wet, semi-dry and dry methods is presented together with MATLAB-based feature extraction tools and R-based modeling pipelines for PLS-DA. The dataset includes raw and preprocessed spectral matrices, as well as feature-extracted datasets generated from the corresponding spectra. The associated computational tools allow users to assess full-spectrum and feature-extracted modeling approaches, analyze green coffee, roasted coffee or both stages simultaneously, and compare classification performance under different preprocessing conditions. This dataset provides a reproducible basis for future studies focused on coffee authentication, non-destructive quality control, chemometric classification and data-driven spectral analysis in agri-food systems.

## 3 Data Description

The experimental dataset was compiled into six Excel files, eight MATLAB scripts, and one R script. It includes raw and preprocessed mid-infrared spectra, MATLAB-based feature extraction routines, and R-based data-driven PLS-DA tools for the multivariate classification of coffee samples processed by wet, semi-dry, and dry post-harvest methods. The computational pipeline allows users to evaluate different modeling scenarios according to the spectral preprocessing strategy, either full-spectrum or feature-extracted information, and the coffee stage considered, including green coffee, roasted coffee, or both stages simultaneously.

**RawMidSpectralCoffeeProcessed.xlsx:** This Excel file contains the raw Fourier transform mid-infrared spectral dataset of green and roasted specialty coffee samples processed by three post-harvest methods: wet, semi-dry and dry processing. The file includes two sheets: *Raw* and *FeatureExtractedRaw*. The Raw sheet contains the full-spectrum dataset. It includes 162 individual coffee samples organized in rows. The first five columns correspond to categorical dummy variables. The columns Wet, Semi-dry and Dry identify the post-harvest processing method of each sample, while the columns Green and Roasted identify the coffee processing stage. The remaining columns contain the raw spectral absorbance values recorded across the mid-infrared region, covering approximately 4000-650 cm^–1^. Each spectral column represents the absorbance at a specific wavenumber, and each row represents one coffee sample. The dataset includes a balanced structure with 54 samples per post-harvest processing method and 81 samples for each coffee stage, green and roasted. The *FeatureExtractedRaw* sheet contains the same categorical descriptors, together with six spectral features extracted from the raw spectra: INT, ENG, STD, SKE, KURT and ENT. These variables represent spectral integral, energy, standard deviation, skewness, kurtosis and entropy, respectively. These features were computed used the MATLAB script **FeatureExtraction.m** (see details below), enabling the possibility to compare a modeling strategy based full-spectrum using raw absorbance data and feature-extracted modeling based on these statistical parameters computed from the original spectra.

Raw mid-infrared spectral data was subsequently preprocessed via baseline-correction, SNV, MSC, D1 and D2. This process was conducted using the computer-aided method for preprocessing mid-infrared, near-infrared and Raman-infrared spectral data reported in https://data.mendeley.com/datasets/gcf9cz6hsr/2. This R-based tools allowed us to preprocess our mid-infrared spectra using a validated framework. Therefore, the description of SNV, MSC, D1 and D2 preprocessed datasets are described below:

**BaselineMidSpectralCoffeeProcessed.xlsx:** This Excel file contains the baseline-corrected mid-infrared spectral dataset of green and roasted specialty coffee samples processed by wet, semi-dry and dry methods. The file is organized into two sheets: *Baseline* and *FeatureExtractedBaseline*. The Baseline sheet contains the full spectral matrix after baseline correction. The first five columns correspond to the sample descriptors used for classification and stage identification. The variables Wet, Semi-dry and Dry define the post-harvest processing class, whereas Green and Roasted define the coffee stage. The remaining columns contain baseline-corrected absorbance values for the wavenumber range approximately 4000-650 cm^–1^. Each row corresponds to an individual sample and each spectral column corresponds to a specific wavenumber. The *FeatureExtractedBaseline* sheet contains the same class and stage descriptors, followed by the feature-extracted variables INT, ENG, STD, SKE, KURT and ENT, calculated from the baseline-corrected spectra. This file allows users to evaluate whether removing baseline drift improves the discrimination of coffee samples according to post-harvest processing method, either using the complete spectral matrix or the reduced set of spectral parameters [17].

**SNVMidSpectralCoffeeProcessed.xlsx:** This Excel file contains the SNV preprocessed mid-infrared spectral dataset of green and roasted specialty coffee samples obtained from wet, semi-dry and dry post-harvest processing methods. The file includes two sheets: *SNV* and *FeatureExtractedSNV*. The SNV sheet contains the full-spectrum dataset after SNV preprocessing. The first five columns provide the categorical information required for model construction and interpretation. The variables Wet, Semi-dry and Dry identify the post-harvest processing method, while Green and Roasted indicate whether each sample corresponds to green or roasted coffee. The remaining columns contain the preprocessed absorbance values distributed across the mid-infrared spectral region from approximately 4000 to 650 cm^–1^. The *FeatureExtractedSNV* sheet provides the same categorical descriptors together with the six numerical parameters extracted from the SNV-preprocessed spectra: INT, ENG, STD, SKE, KURT and ENT. This file is suitable for assessing the effect of scatter and intensity normalization on the classification of coffee samples, enabling both full-spectrum and feature-extracted partial least squares discriminant analysis modeling.

**MSCMidSpectralCoffeeProcessed.xlsx:** This Excel file contains the MSC preprocessed mid-infrared spectral dataset of green and roasted specialty coffee samples classified according to wet, semi-dry and dry post-harvest processing methods. The file contains two sheets: *MSC* and *FeatureExtractedMSC*. The MSC sheet includes the full-spectrum matrix after multiplicative scatter correction. The first five columns are categorical dummy variables. The columns Wet, Semi-dry and Dry encode the post-harvest processing method, whereas Green and Roasted encode the coffee stage. The remaining spectral columns contain absorbance values corrected for multiplicative scattering effects over the wavenumber range approximately 4000-650 cm^–1^. The *FeatureExtractedMSC* sheet contains the same categorical information and the six extracted spectral parameters INT, ENG, STD, SKE, KURT and ENT, calculated from the MSC-preprocessed spectra. This file allows researchers to investigate whether scatter-corrected spectral fingerprints improve post-harvest process discrimination and to compare the performance of complete spectral profiles against compact feature-based representations.

**D1MidSpectralCoffeeProcessed.xlsx:** This Excel file contains the D1 mid-infrared spectral dataset of green and roasted specialty coffee samples processed using wet, semi-dry and dry post-harvest methods. The file is composed of two sheets: *D1* and *FeatureExtractedD1*. The D1 sheet contains the full-spectrum matrix after first-derivative preprocessing. The first five columns contain the categorical descriptors of the dataset. The columns Wet, Semi-dry and Dry indicate the post-harvest processing method, while Green and Roasted identify the coffee processing stage. The remaining columns contain first-derivative spectral values across the mid-infrared wavenumber range of approximately 4000-650 cm^–1^. First-derivative preprocessing is useful for enhancing spectral resolution and reducing baseline-related effects, making this dataset suitable for evaluating subtle spectral differences associated with processing method and roasting stage. The *FeatureExtractedD1* sheet contains the same categorical descriptors and the six feature-extracted variables obtained from the first-derivative spectra: INT, ENG, STD, SKE, KURT and ENT. This file enables classification modeling based on either the complete first-derivative spectral profile or derivative-derived statistical descriptors.

**D2MidSpectralCoffeeProcessed.xlsx:** This Excel file contains the second-derivative mid-infrared spectral dataset of green and roasted specialty coffee samples obtained from wet, semi-dry and dry post-harvest processing methods. The file includes two sheets: *D2* and *FeatureExtractedD2*. The D2 sheet contains the full second-derivative spectral matrix. The first five columns correspond to the classification and stage descriptors. The variables Wet, Semi-dry and Dry define the post-harvest processing method, whereas Green and Roasted define the coffee stage. The remaining columns contain second-derivative spectral values for the wavenumber region approximately 4000-650 cm^–1^. Second-derivative preprocessing can enhance overlapping spectral bands and improve the detection of subtle chemical differences among samples. The *FeatureExtractedD2* sheet contains the same categorical descriptors together with the six extracted spectral features INT, ENG, STD, SKE, KURT and ENT, calculated from the second-derivative spectra. This file supports the evaluation of high-resolution derivative-based spectral information for discriminating specialty coffee according to post-harvest processing method.

In addition to the full-spectrum and global feature-extraction datasets, each of the six Excel files YY contains two supplementary worksheets designed to investigate the discriminatory potential of selected mid-infrared spectral regions. Three spectral intervals were considered: 1800-1700 cm^–1^, encompassing the ester C=O stretching band near 1742 cm^–1^; 1650-1500 cm^–1^, including the amide II band near 1541 cm^–1^; and 1200-900 cm^–1^, corresponding to the carbohydrate fingerprint region. These intervals were selected based on their chemical relevance and potential contribution to the discrimination of coffee post-harvest processing methods. Their inclusion also addresses a limitation of global feature extraction: statistical features such as skewness and kurtosis can be strongly influenced by prominent spectral signals, including the broad O–H stretching band near 3260 cm^–1^ and the intense C–O stretching band near 1027 cm^–1^, potentially biasing the extracted features toward these dominant spectral contributions. To investigate these regions, two additional predictor representations were incorporated into the dataset. The first retains the original spectral variables within the three predefined intervals while excluding the remaining wavenumbers, enabling PLS-DA modeling using full-spectrum data restricted to specific spectral regions. The second applies the MATLAB-based feature-extraction procedure (**See FeatureExtraction_SpecificSpectralRegion.m**) independently to each interval, calculating six quantitative features (INT, ENG, STD, SKE, KURT, and ENT) for each region. This produces 18 region-specific features and enables PLS-DA modeling using features extracted from predefined spectral regions. Together, these supplementary datasets facilitate the assessment of whether restricting the predictor space to chemically relevant spectral intervals, either through direct spectral variables or region-specific feature extraction, improves the discrimination of coffee according to its post-harvest processing method.

Given that the discrimination of green and roasted specialty coffee according to post-harvest processing method relies on data-driven modeling, the choice of spectral representation is a critical consideration. The original full-spectrum dataset comprises 1,798 wavenumber variables, whereas global feature extraction reduces this high-dimensional representation to six quantitative features (INT, ENG, STD, SKE, KURT, and ENT). Although this substantial dimensionality reduction may simplify model development, it may also result in the loss of localized spectral information relevant to class discrimination. Therefore, its effect on classification performance warrants systematic investigation. To address this issue, the full-spectrum data were also restricted to three predefined spectral regions (1800-1700, 1650-1500, and 1200-900 cm^–1^), reducing the predictor space from 1,798 to 299 wavenumber variables while retaining spectral information from chemically relevant bands. In addition, the same six spectral features were computed independently within each of the three predefined regions, generating 18 region-specific features in total, that is, six features per region. Accordingly, four complementary predictor representations were considered: (i) full-spectrum data (1,798 wavenumbers), (ii) full-spectrum data restricted to predefined spectral regions (299 wavenumbers), (iii) features extracted from the entire spectrum (6 variables), and (iv) features extracted from predefined spectral regions (18 variables). This framework enables a systematic comparison of the discriminatory performance of different spectral representations and an assessment of the trade-off between dimensionality reduction and the retention of information relevant to coffee post-harvest processing classification.

Beyond the four spectral representations described above, wavelet-based transformation offers an additional strategy for investigating the discriminatory information contained in mid-infrared spectra. Wavelet analysis enables spectral signals to be examined at different scales, providing an alternative means of characterizing localized spectral variations that may not be adequately captured by conventional full-spectrum modeling or global feature extraction. This approach can be investigated using either the entire spectrum or predefined spectral regions, followed by the extraction of quantitative features from the transformed signals or their wavelet coefficients. Such combinations provide opportunities to assess how wavelet selection and feature extraction influence the retention of information relevant to coffee post-harvest processing discrimination.

To facilitate further investigation, the dataset is accompanied by MATLAB routines for wavelet-based spectral feature extraction, including **FeatureExtraction_SpecificSpectralRegionWavelet.m**, together with the feature-calculation functions **Integral.m, energy.m, Stds.m, Skew.m, Kurt.m**, and **Entrop.m**. An illustrative application of the discrete wavelet transform (DWT), using the Daubechies wavelet of order four (db4), is also provided in the **WaveletSpectralAnalysis.xlsx Excel file.** This implementation serves as an example rather than an exhaustive assessment of wavelet-based representations. Other Daubechies wavelets, decomposition levels, and alternative wavelet families, as well as continuous wavelet transform (CWT) approaches, can be explored in further studies. Accordingly, the provided dataset and computational routines establish a basis for investigating whether wavelet-based spectral representations, either independently or combined with feature extraction, can improve the characterization and discrimination of coffee according to its post-harvest processing method.

The mid-infrared spectra of green and roasted specialty coffee samples processed by W, SD and Dr methods showed the main vibrational regions associated with the chemical composition of coffee beans (Fig. 1). As observed in the raw spectra, differences between green and roasted samples were mainly reflected in changes in absorbance intensity and band shape across the 4000-650 cm^–1^ region. The broad band around 3260 cm^–1^ can be associated with O-H stretching vibrations, mainly related to water, carbohydrates and phenolic compounds [18]. The bands observed at 2922 and 2853 cm^–1^ correspond to symmetric and asymmetric C-H stretching vibrations, commonly associated with lipids, aliphatic chains and other organic compounds present in coffee [19]. The signal around 1742 cm^–1^ can be attributed to C=O stretching vibrations of ester groups, while the region near 1638 cm^–1^ may be related to water bending, C=C stretching or protein-associated vibrations. Additional bands at 1541, 1459 and 1377 cm^–1^ are linked to N–H, C–H and CH_2_/CH_3_ deformation vibrations, whereas the fingerprint region between 1241 and 1027 cm^–1^ is mainly associated with C–O, C–O–C and C–N vibrations from carbohydrates, proteins and other matrix components. The bands below 900 cm^–1^, including those located at 870, 810, 762, 712 cm^–1^, may be related to aromatic and out-of-plane deformation vibrations. These spectral differences suggest that both post-harvest processing and roasting modify the chemical fingerprint of the coffee samples.

**Fig. 1:**
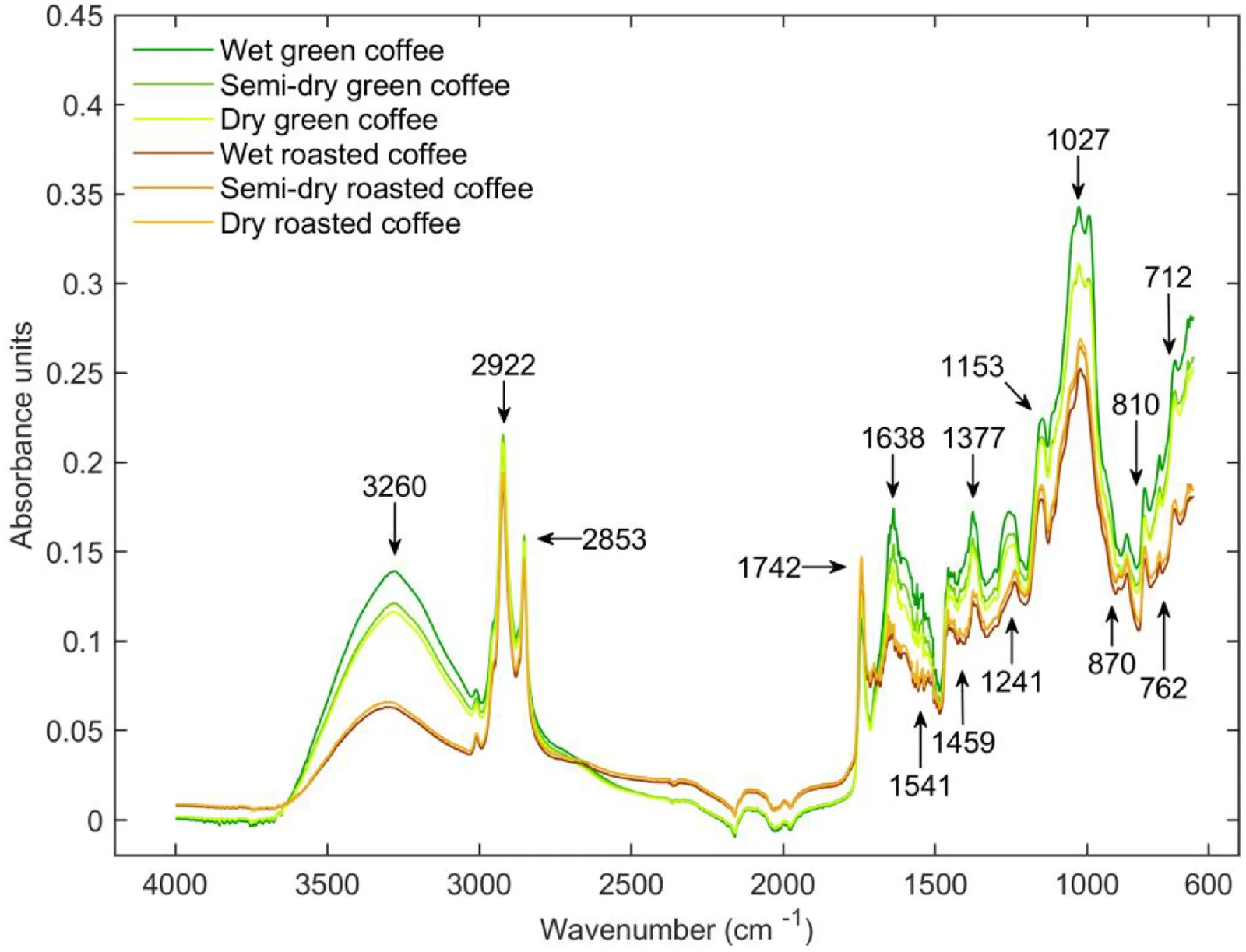
Raw mid-infrared spectra of green and roasted specialty coffee samples processed by wet, semi-dry and dry post-harvest methods. The spectra showed the main absorption bands across the 4000-650 cm^–1^ region, with their wavenumbers corresponding to characteristic vibrational regions of coffee constituents. Differences in band intensity and spectral shape between green and roasted samples, as well as among post-harvest processing methods, indicate changes in the chemical fingerprint associated with processing and roasting. These spectral profiles were used as the basis for subsequent preprocessing, feature extraction and classification modeling.

Qualitative comparison of the spectral profiles reveals that differences between green and roasted coffee are generally more pronounced than those among post-harvest processing methods. Green coffee samples exhibit comparatively higher absorbance around 3260 cm^–1^ and at several fingerprint-region bands, particularly near 1638, 1377, 1153, 1027 and 712 cm^–1^, whereas roasted samples show a comparatively prominent band near 1742 cm^–1^. This consideration is particularly relevant to PLS-DA modeling, as models incorporating spectra from both green and roasted coffee may predominantly capture the pronounced spectral variability associated with roasting, potentially masking the subtler discriminant information related to post-harvest processing methods (W, SD and Dr). To address this issue, the proposed modeling framework (see **PLSDA_Modeling_Pipelines_Advanced.R**) allows the calibration of stage-specific models using green or roasted coffee samples independently, as well as combined models incorporating both coffee stages. Nevertheless, the relative suitability of these strategies cannot be established solely through visual inspection of the spectra and must be assessed based on models and experimental data. Accordingly, the modeling framework enables a systematic investigation of different modeling configurations, including coffee-stage selection, spectral preprocessing, predictor representation (full-spectrum or feature-extracted variables), training-validation partitioning, and classification scenarios involving either the three processing methods simultaneously or their pairwise binary combinations. Model performance is assessed through training, cross-validation and independent external validation, allowing the discriminatory information associated with post-harvest processing to be examined under different analytical conditions.

Regarding the differences within the green coffee group (Fig. 1), W-processed samples generally exhibit higher absorbance at several of these bands, while the SD- and Dr-processed spectra display closely similar profiles. Likewise, the spectra of roasted W, SD and Dr samples largely overlap, with differences mainly associated with variations in relative band intensity and shape rather than the appearance of distinct processing-specific absorption bands. These spectral differences suggest that both post-harvest processing and roasting modify the chemical fingerprint of the coffee samples. However, the spectral variability observed across the samples should not be interpreted as evidence of complete discrimination among the three post-harvest processing methods. While visual inspection reveals differences associated with the overall chemical composition of green and roasted coffee, W, SD and Dr samples share most of their characteristic absorption bands, making their identification from individual spectral features difficult. This apparent contrast arises because spectral variability and class discrimination represent different aspects of the data: the former reflects differences in the measured chemical fingerprints, whereas the latter requires those differences to be sufficiently systematic and processing-specific to distinguish the predefined classes. Multivariate modeling is therefore necessary to evaluate the combined contribution of multiple, potentially correlated spectral variables and identify discriminant patterns that may not be evident from visual inspection of individual bands.

The preprocessed spectra (Fig. 2) showed how each spectral treatment modified the original infrared information before mathematical modeling. The raw spectra displayed differences in intensity and baseline behavior among samples, while baseline correction reduced background effects and improved the comparability of spectral profiles. SNV and MSC minimized intensity dispersion and scattering-related variability, generating more homogeneous spectral patterns. The D1 and D2 derivative spectra enhanced narrow bands and subtle spectral variations, especially in the fingerprint region, although with lower signal amplitude and sharper positive and negative peaks. Furthermore, the visual inspection of the spectra revealed relevant chemical variability among coffee samples; however, the overlap between spectral profiles makes it difficult to clearly distinguish wet, semi-dry and dry processed samples only through qualitative analysis. Therefore, multivariate modeling using full-spectrum and/or feature-extracted PLS-DA was required to better assess the discriminatory information contained in the mid-infrared spectral dataset.

**Fig. 2:**
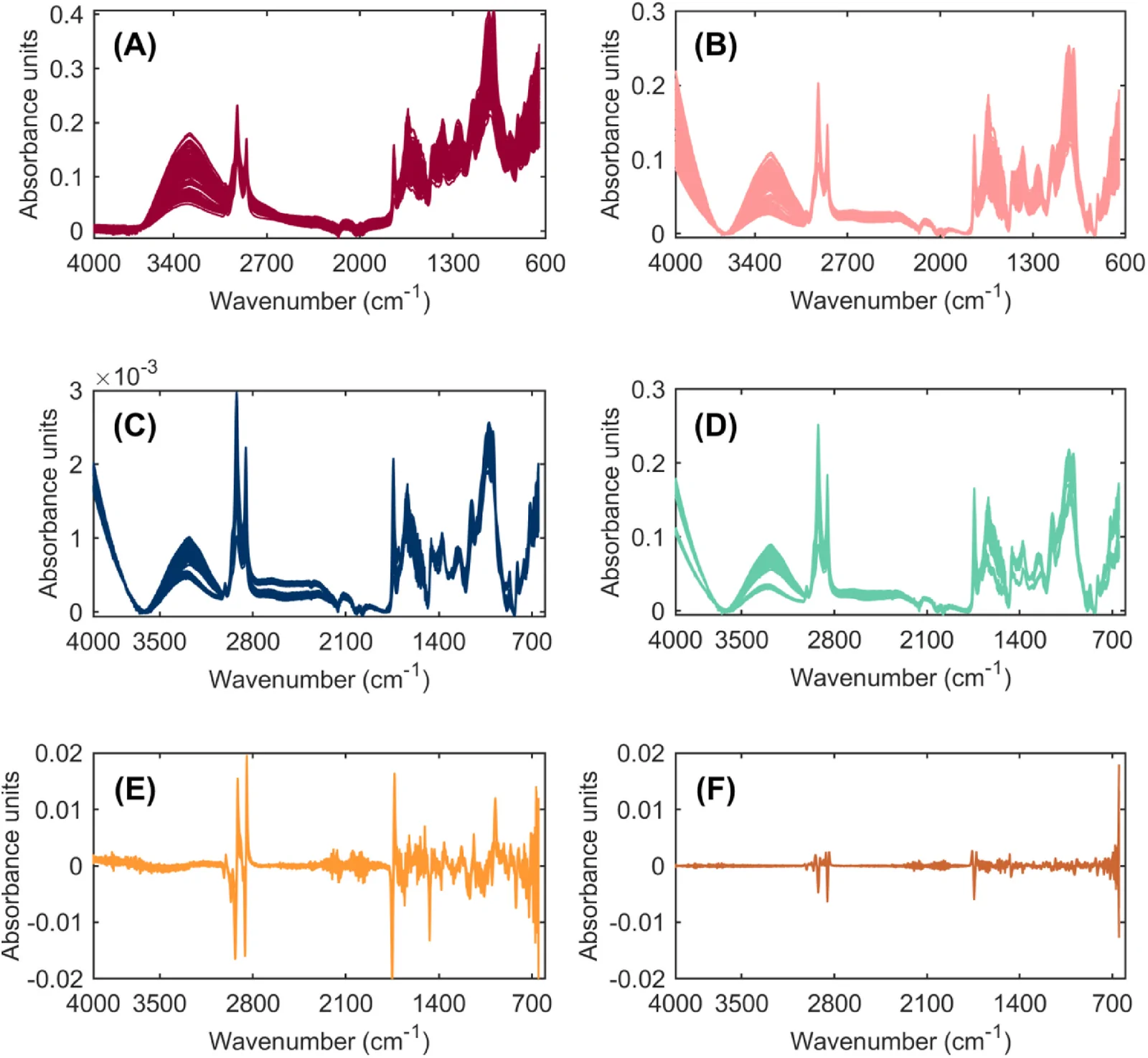
Raw and preprocessed mid-infrared spectra of green and roasted specialty coffee samples used for chemometric modeling. The spectral dataset is shown after different preprocessing strategies: raw spectra (A), baseline-corrected spectra (B), standard normal variate spectra (C), multiplicative scatter correction spectra (D), first-derivative spectra (E), and second-derivative spectra (F). These preprocessing treatments were applied to reduce baseline variation, correct scattering effects, normalize spectral intensity differences, and enhance subtle spectral features before full-spectrum and feature-extracted partial least squares discriminant analysis modeling.

**FeatureExtraction.m**: This MATLAB scripts provide a modular feature-extraction workflow for extracting relevant parameters on raw and preprocessed mid-infrared spectral datasets into a numerical variables suitable for subsequent chemometric modeling. This script acts as the main execution file. First, it loads the file **dataPreprocessedSpectra.mat**, which contains the preprocessed spectral matrices (above described), and iterates sample by sample across the available spectral datasets. For each spectrum, it extracts six statistical and signal-related variables: INT, ENG, STD, SKE, KURT and ENT. This procedure is applied independently to the raw, baseline-corrected, SNV, MSC, D1 and D2 spectral matrices, generating a feature dataset for each preprocessing strategy. **FeatureExtraction.m** is therefore the coordinating script that links all auxiliary MATLAB functions. For each spectral row, it calls **Integral.m, Energy.m, Stds.m, Skew.m, Kurt.m** and **Entrop.m** in a fixed sequence, storing their outputs as columns of the corresponding feature matrix. In this workflow, the first column represents spectral integral, the second column represents spectral energy, the third column represents spectral standard deviation, the fourth column represents spectral skewness, the fifth column represents spectral kurtosis and the sixth column represents spectral entropy. This same feature structure is maintained for all preprocessing strategies, allowing direct comparison between raw spectra and preprocessed spectra during subsequent partial least squares discriminant analysis modeling.

The **Integral.m** function calculates the integrated spectral response. In the direct extraction mode used by **FeatureExtraction.m**, negative intensity values are set to zero and the integral is computed using trapezoidal numerical integration [20]. This descriptor summarizes the total spectral intensity or global absorbance contribution of each spectrum. The function also includes an alternative wavelet-based extraction mode, in which the signal is decomposed using Daubechies wavelets and the third-level detail coefficients are integrated after removing negative values.

The **Energy.m** function calculates the spectral energy as the squared Euclidean norm of the input spectrum [20]. This descriptor quantifies the overall magnitude of the spectral signal and is sensitive to global differences in absorbance intensity. In its wavelet-based mode, the same energy calculation is applied to the third-level detail coefficients obtained after wavelet decomposition, providing a frequency-localized representation of signal energy.

The **Stds.m** function computes the standard deviation of the spectral signal [21]. This variable describes the dispersion of absorbance values across the spectral range and provides information about the variability of the spectral profile. In the wavelet-based branch, the standard deviation is calculated from the third-level detail coefficients, allowing the variability of localized spectral fluctuations to be quantified.

The **Skew.m** function extracts the skewness of the spectral signal [12]. This descriptor quantifies the asymmetry of the absorbance distribution along the spectrum and may capture differences in the relative contribution of high- and low-intensity spectral regions. When the wavelet option is selected, skewness is computed from the third-level detail coefficients, providing an asymmetry descriptor of the decomposed spectral component.

The **Kurt.m** function calculates the kurtosis of the spectral signal [12]. This descriptor provides information on the peakedness and tail behavior of the spectral intensity distribution. In the context of mid-infrared spectra, kurtosis can help summarize whether the spectral profile is dominated by sharper bands or by a more broadly distributed absorbance structure. The function also supports wavelet-based kurtosis extraction from the third-level detail coefficients.

The **Entrop.m** function calculates the entropy of the spectral signal [12]. This descriptor summarizes the degree of spectral complexity or disorder and may be useful for capturing changes in the distribution of absorbance information across the spectral profile. In the alternative wavelet-based mode, entropy is obtained from the third-level detail coefficients after wavelet decomposition, enabling the characterization of localized spectral complexity.

After completing feature extraction, **FeatureExtraction.m** also generates a six-panel visualization of the spectral matrices used in the analysis. The plots correspond to raw, baseline-corrected, SNV, MSC, D1 and D2 spectra (Fig. 2). The wavenumber axis is displayed in reverse order, which is consistent with conventional mid-infrared spectral representation. This graphical section provides a visual comparison of the spectral structure after each preprocessing strategy and supports the interpretation of how preprocessing modifies the spectral profiles before feature extraction and classification modeling.

The **FeatureExtraction_SpecificSpectralRegion.m** script extends the MATLAB feature-extraction workflow by calculating quantitative spectral features independently within three predefined mid-infrared regions rather than across the entire spectrum. The script first loads **dataPreprocessedSpectra.mat**, which contains the raw and preprocessed spectral matrices, and processes the raw, baseline-corrected, SNV, MSC, D1, and D2 datasets independently. For each sample, the three selected spectral regions are extracted using the predefined column ranges 135:296 for 1200-900 cm^–1^ (Region 1; R1), 457:537 (Region 2; R2: 1650-1500 cm^–1^), and 565:618 (Region 3; R3: 1800-1700 cm^–1^). The six features (INT, ENG, STD, SKE, KURT and ENT) are then calculated separately within each region using the corresponding auxiliary MATLAB functions in their direct mode. This procedure generates six features per spectral region, resulting in 18 region-specific features for each sample. The resulting variables are organized into six groups, each containing the three regional values of a given feature (e.g., INT_R1, INT_R2, and INT_R3). The same feature structure is maintained across all six preprocessing strategies. This approach provides a reduced-dimensional representation of localized spectral characteristics, facilitating the investigation of chemically relevant absorption regions that may be less apparent when features are calculated across the entire spectrum.

The **FeatureExtraction_SpecificSpectralRegionWavelet.m** script provides a wavelet-based extension of the region-specific feature-extraction procedure. It loads **dataPreprocessedSpectra.mat** and processes the raw, baseline-corrected, SNV, MSC, D1, and D2 spectral matrices independently. For each sample, the same three predefined spectral regions (R1, R2 and R3) are selected. Unlike the direct extraction approach, this script calls the auxiliary feature-calculation functions (**Integral.m, Energy.m, Stds.m, Skew.m, Kurt.m and Entrop.m)** with the arguments (1,4), activating their wavelet-based processing mode. The wavelet-based procedure is applied independently to each spectral region, followed by the calculation of INT, ENG, STD, SKE, KURT, and ENT from the corresponding transformed spectral information. As in the direct extraction workflow, six features are calculated for each of the three spectral regions, yielding 18 wavelet-based region-specific features per sample.

**PLSDA_Modeling_Pipelines_Advanced.R**: This R script provides an interactive and reproducible computational workflow for PLS-DA of specialty coffee samples subjected to W, SD, and Dr post-harvest processing. The pipeline was developed to investigate how spectral preprocessing, predictor representation, coffee stage, and classification configuration influence the discrimination of post-harvest processing methods. Unlike workflows restricted to a single spectral representation, the script accommodates four complementary modeling approaches: (i) full-spectrum data, (ii) full-spectrum data restricted to predefined spectral regions, (iii) features extracted from the entire spectrum, and (iv) features extracted from predefined spectral regions. This structure facilitates the comparison of high-dimensional spectral information with reduced-dimensional representations obtained through spectral-region selection and quantitative feature extraction.

The script first establishes the computational environment by checking, installing, and loading the required R packages for data handling, classification, visualization, model validation, and results export. PLS-DA modeling is implemented using the *ropls* package [22], whereas *caret* supports stratified data partitioning and classification performance assessment. Additional packages, including *readxl, openxlsx, ggplot2*, and *ggrepel*, provide data import, numerical export, and graphical visualization functionalities. This organization allows the analytical procedure to be reproduced using the accompanying datasets and computational resources. The data-loading module is interactive and initially prompts the user to select one of six spectral preprocessing strategies: raw spectra, baseline correction, SNV, MSC, D1 or D2. Each preprocessing option is associated with its corresponding Excel file, while the selected modeling approach determines the worksheet imported for analysis. Consequently, the user can develop a PLS-DA model using the complete spectral matrix, spectral variables restricted to predefined regions, six features extracted from the entire spectrum (INT, ENG, STD, SKE, KURT, and ENT), or 18 features obtained by calculating these six parameters independently within each of the three predefined spectral regions. Before data import, the script verifies the existence of the selected file in the working directory and reports the corresponding dataset dimensions. This procedure provides a consistent framework for investigating the influence of spectral preprocessing and predictor representation under comparable modeling conditions.

Following data import, the user defines the coffee stage to be considered during model development. Three alternatives are available: green coffee only, roasted coffee only, or green and roasted coffee simultaneously. Stage-specific modeling enables the discrimination of post-harvest processing methods within a particular coffee state, whereas the combined-stage configuration allows the model to be developed using spectral observations from both green and roasted samples. The script applies the corresponding sample-selection procedure and reports the number of observations retained for subsequent analysis.

The categorical response variable, designated Process, is constructed from the original dummy-coded variables corresponding to W, SD, and Dr processing. The user can subsequently define either a multiclass discrimination problem involving all three processing methods or one of three binary classification scenarios: W vs. Dr, W vs. SD, or SD vs. Dr. For binary classification, observations belonging to the excluded class are removed, and unused factor levels are discarded. Thus, the classification problem can be tailored to the analytical objective without requiring modifications to the underlying modeling code.

The predictor matrix is defined automatically according to the selected spectral representation. For full-spectrum approaches, the script retains the numeric spectral variables available in the corresponding worksheet. For feature-extraction approaches, it selects either the six features calculated from the entire spectrum or the available region-specific features. Importantly, the dummy-coded processing variables, categorical response, coffee-stage indicators, and designated metadata fields are excluded from the predictor matrix. Therefore, the model is calibrated using spectral information rather than directly incorporating variables that identify the target processing class or coffee stage. Observations containing missing values in the selected modeling variables are removed, and zero-variance predictors are excluded before calibration. The script subsequently reports the final number of samples, number of predictors, and variables retained for analysis.

The resulting dataset is partitioned into training and holdout validation subsets using stratified sampling based on the processing class. The user can select training proportions of 70%, 75%, or 80%, with the remaining 30%, 25%, or 20% reserved for holdout validation, respectively. A fixed random seed is used to support reproducibility. Model calibration is performed on the training subset using *ropls*::opls(), with standard scaling, five-fold cross-validation, 20 permutation tests, and no orthogonal components, thereby retaining a conventional PLS-DA structure. The optimal number of predictive latent variables (NPLS) is automatically determined by identifying the first local maximum in classification accuracy obtained through five-fold cross-validation within the training subset. The selected number of latent variables is subsequently used to calibrate the final PLS-DA model, which is saved as an R Data Serialization (RDS) file for reproducible reuse.

To provide a more comprehensive assessment of classification performance, the pipeline distinguishes three evaluation stages: training, K-fold out-of-fold cross-validation-OOF-CV, and holdout validation. In addition to the internal cross-validation performed by *ropls*, an explicit stratified five-fold OOF-CV procedure is implemented within the training subset. During each iteration, a separate PLS-DA model is calibrated using four folds and applied to the excluded fold. The predictions obtained across all five iterations are subsequently combined, ensuring that each training observation receives a prediction from a model in which that observation was not included during calibration. These predictions are used to calculate cross-validated classification metrics and construct an independent OOF confusion matrix, without using the reserved holdout validation subset. The pipeline also extracts model-quality statistics from the *ropls* summary, including cumulative explained variation in the predictor matrix (R^2^X), cumulative explained variation in the response (R^2^Y), cumulative cross-validated predictive ability (Q^2^), and root mean square error of estimation (RMSE). These statistics complement the classification metrics derived from the observed and predicted class labels. In particular, Q^2^ originates from the internal cross-validation implemented by *ropls*, whereas the OOF classification metrics are calculated explicitly from the predictions obtained across the five folds.

Following calibration and cross-validation, the fitted PLS-DA model is applied independently to the training and holdout validation datasets. The predicted classes are compared with their reference labels to generate three confusion matrices corresponding to training, OOF-CV, and holdout validation. Moreover, the classification performance of PLSDA model is quantified using accuracy, Cohen’s kappa, sensitivity, and specificity. For consistency across binary and multiclass configurations, sensitivity and specificity are calculated separately for each processing class using a one-versus-all. In addition, the script reports class-specific sensitivity, specificity, positive predictive value, negative predictive value, prevalence, detection rate, detection prevalence, and balanced accuracy for each evaluation stage. This reporting structure enables the examination of both predictive performance and class-dependent misclassification patterns.

The graphical module generates training and holdout validation confusion matrices, together with PLS-DA score plots representing the first two latent variables (LV1 vs. LV2) and, when at least three latent variables are available, LV1 vs. LV3. These score plots include observations colored according to post-harvest processing class and 95% confidence ellipses. Additional visualizations include variable importance in projection (VIP) scores and loading plots, allowing the contribution of individual wavenumbers or extracted features to the fitted model to be investigated. The pipeline also requests the diagnostic plots supported by *ropls*, including score, weight, loading, correlation, outlier, permutation, prediction, and explained-variance representations. Available diagnostic outputs are exported as individual high-resolution images and consolidated PDF files, accompanied by a log documenting their generation status.

To ensure traceability, the script exports the observed and predicted classes, fold assignments, classification metrics, confusion matrices, VIP scores, loadings, and model-quality statistics as individual numerical files. The principal results are additionally consolidated into an Excel file containing separate worksheets for predictions, overall metrics, class-specific metrics, PLS-DA model quality, the *ropls* summary, VIP scores, loadings, and the three confusion matrices. A structured text report (**PLSDA_model_summary**) provides a complementary summary of the selected preprocessing strategy, predictor representation, coffee stage, classification scenario, sample distribution, training-validation partition, model settings, and performance statistics. All numerical outputs and graphical results are automatically saved in a dedicated results directory created within the R working directory. These graphical outputs include training and validation confusion matrices, latent-variable score plots, VIP and loading plots, and the available *ropls* diagnostic visualizations. Figures are exported in high-resolution image formats, while diagnostic plots are additionally consolidated into PDF files and organized in a dedicated subdirectory. This automated output structure ensures that the results of each modeling configuration are systematically organized and readily accessible, facilitating their documentation, interpretation, comparison, and subsequent use in scientific reporting.

Beyond the illustrated configurations, the pipeline enables users to investigate the effects of spectral preprocessing, predictor dimensionality, coffee stage, classification scenario, and training-validation proportion within a consistent computational framework. The inclusion of full-spectrum and region-specific representations, together with global and region-specific feature extraction, provides opportunities to examine the trade-off between dimensionality reduction and the retention of discriminatory spectral information. Furthermore, the explicit separation of training performance, OOF-CV performance, and internal holdout validation supports a more accurate assessment of model performance. The computational workflow may also be adapted to comparable spectral datasets from other agri-food products, providing a basis for multivariate classification, product authentication, and spectroscopic quality assessment.

An additional documentation file, **README_Best_Practice_Guide_PLSDA**, is provided to guide users in the appropriate implementation of the accompanying computational tools. It outlines recommended practices for data preparation, model configuration, validation, and interpretation of PLS-DA results, thereby promoting reproducible and methodologically consistent chemometric analyses.

The illustrative examples of the use of **PLSDA_Modeling_Pipelines_Advanced.R** in the application of the computational workflow to mid-infrared spectra using different examples are illustrated in Fig. 3, Fig. 4, Fig. 5, Fig. 6, Fig. 7. Additionally, the statistical performance results of these models are summarized in Table 1.

**Fig. 3:**
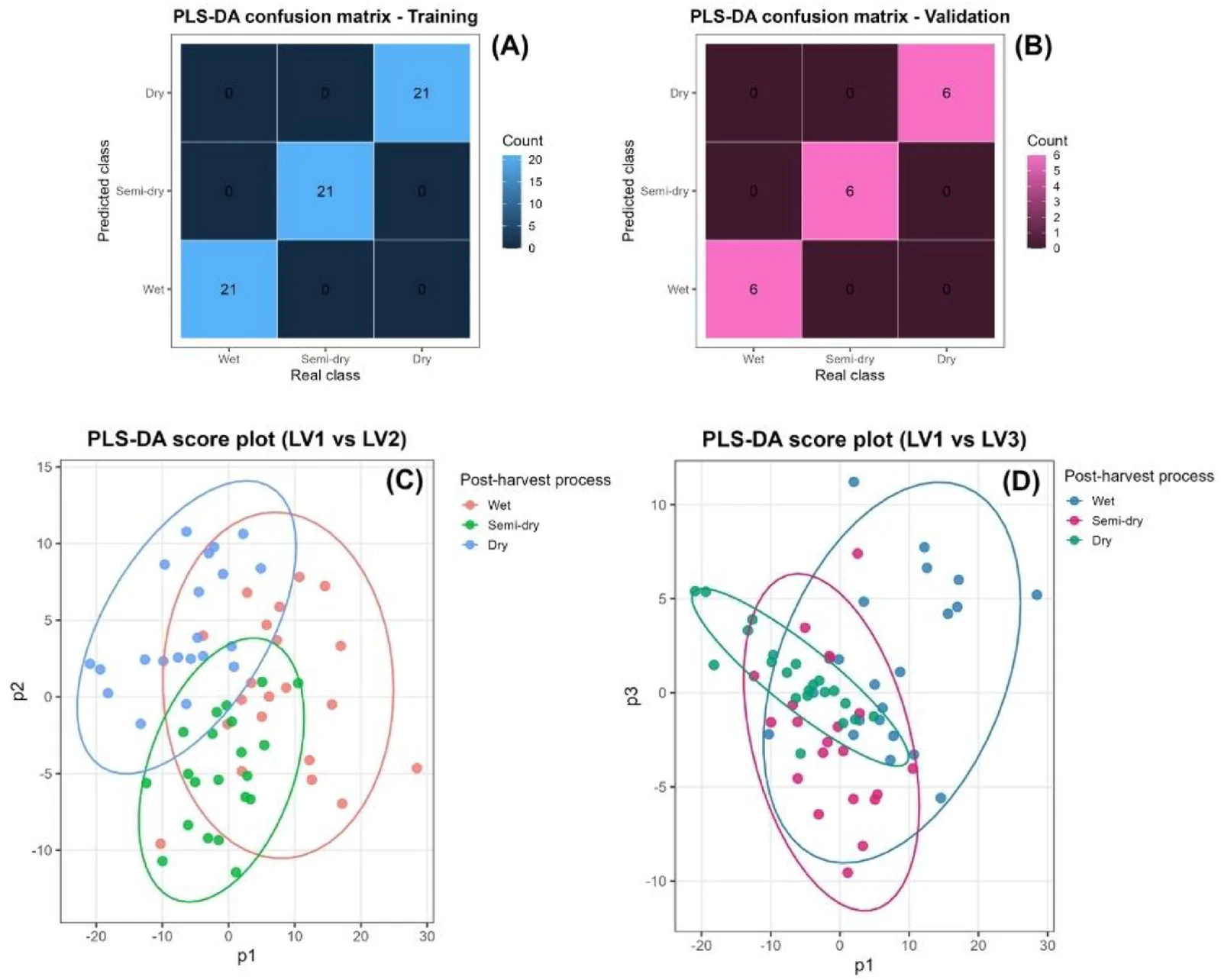
Classification performance and latent-variable score plots of the Partial Least Squares Discriminant Analysis (PLS-DA) model developed using the full-spectrum data restricted to predefined spectral regions approach and green coffee samples for classification according to post-harvest processing method (wet, semi-dry, and dry). Training confusion matrix (A) and holdout validation confusion matrix (B), showing the relationship between observed (horizontal axis) and predicted (vertical axis) classes. Diagonal values represent correctly classified samples, whereas off-diagonal values indicate misclassifications. Score plot of the first and second predictive latent variables (LV1 vs. LV2; C) and score plot of the first and third predictive latent variables (LV1 vs. LV3; D). Samples are colored according to post-harvest processing method, and the ellipses illustrate the distribution of each class within the corresponding latent-variable projection.

**Fig. 4:**
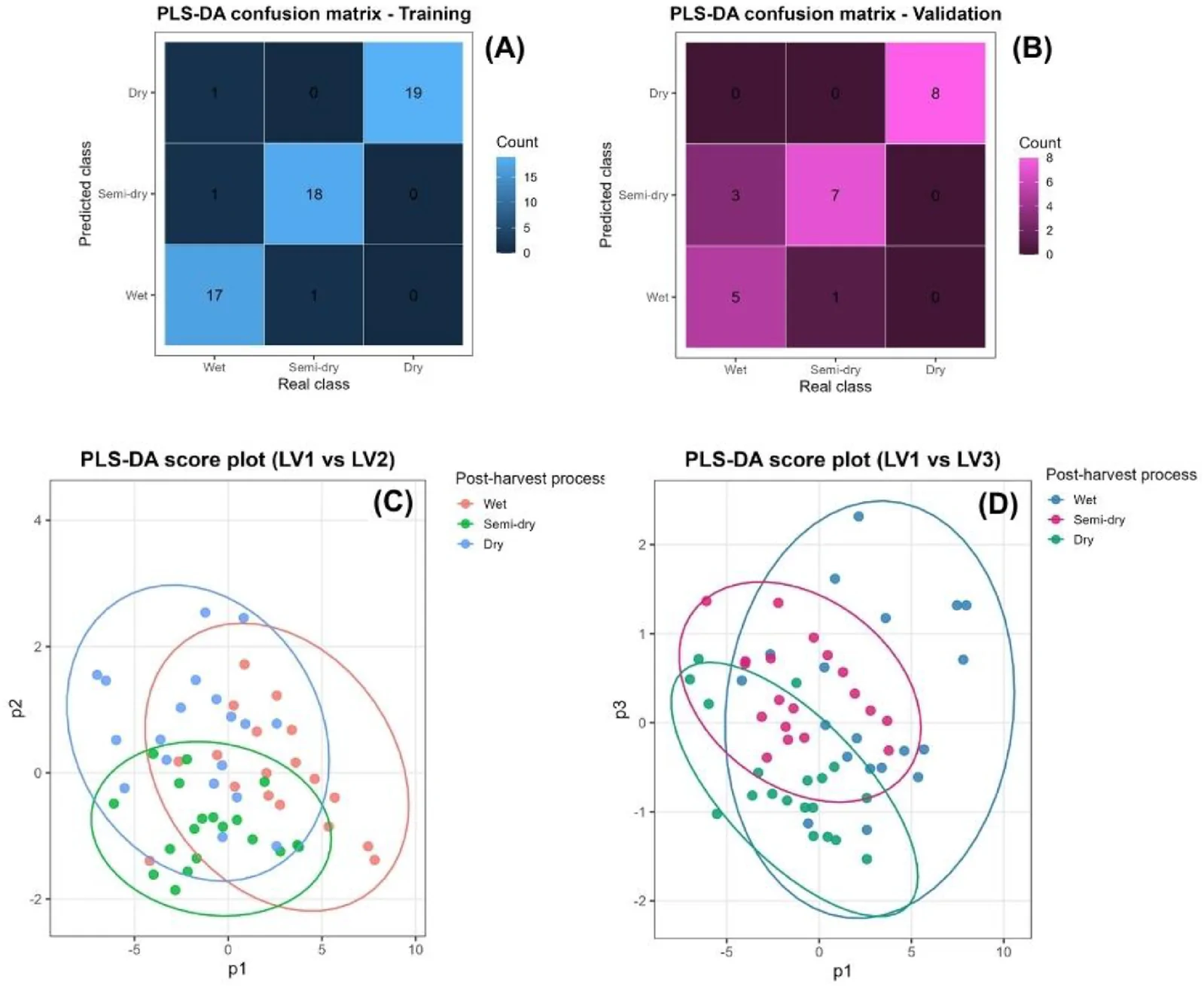
Classification performance and latent-variable score plots of the Partial Least Squares Discriminant Analysis (PLS-DA) model developed using the features extracted from predefined spectral regions and green coffee samples for classification according to post-harvest processing method (wet, semi-dry, and dry). Training confusion matrix (A) and holdout validation confusion matrix (B), showing the relationship between observed (horizontal axis) and predicted (vertical axis) classes. Diagonal values represent correctly classified samples, whereas off-diagonal values indicate misclassifications. Score plot of the first and second predictive latent variables (LV1 vs. LV2; C) and score plot of the first and third predictive latent variables (LV1 vs. LV3; D). Samples are colored according to post-harvest processing method, and the ellipses illustrate the distribution of each class within the corresponding latent-variable projection.

**Fig. 5:**
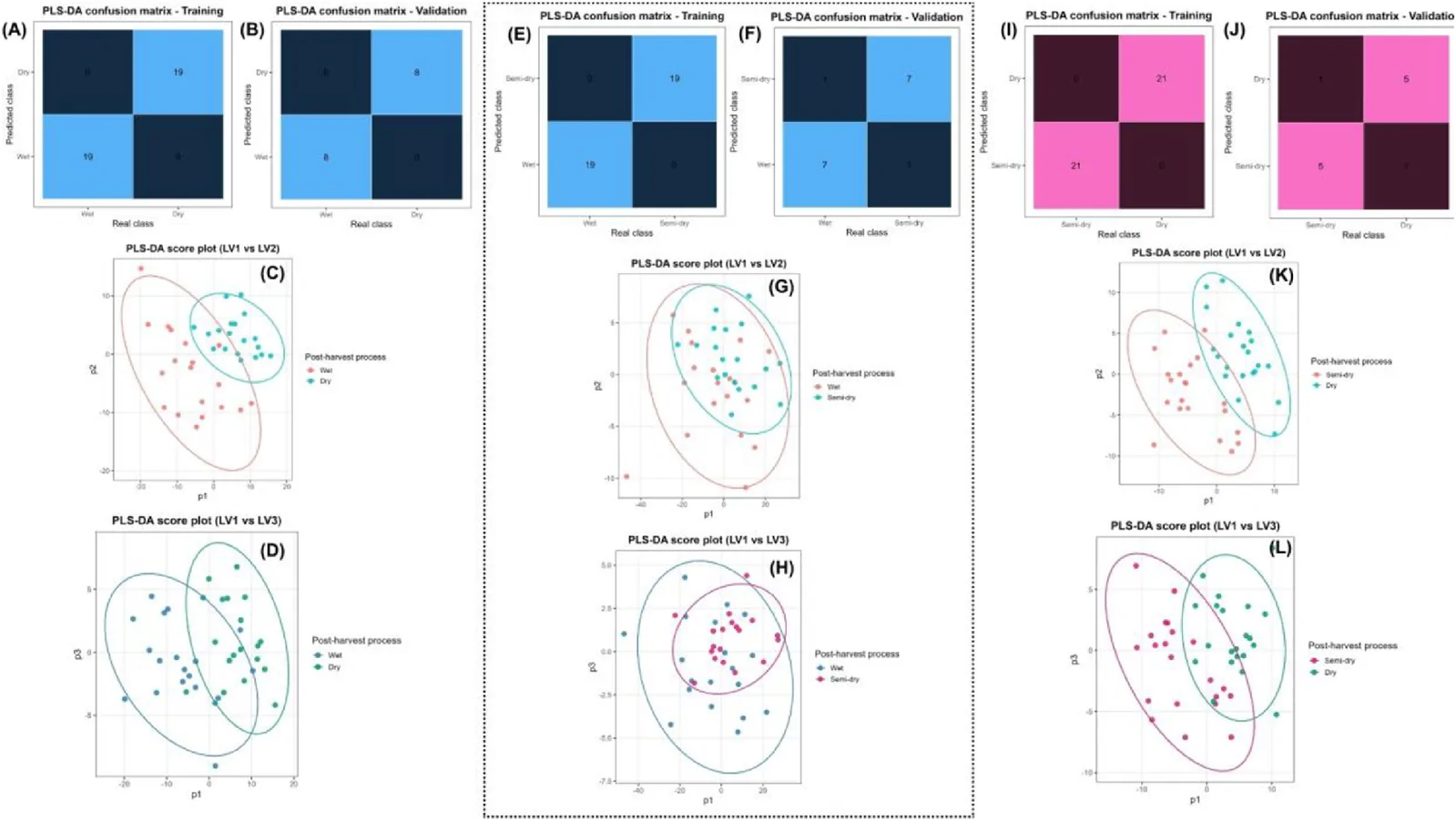
Classification performance and latent-variable score plots of Partial Least Squares Discriminant Analysis (PLS-DA) models developed using the full-spectrum approach and green coffee samples for pairwise classification according to post-harvest processing method. Wet vs. dry classification: training confusion matrix (A), holdout validation confusion matrix (B), score plot of the first and second predictive latent variables (LV1 vs. LV2; C), and score plot of the first and third predictive latent variables (LV1 vs. LV3; D). Wet vs. semi-dry classification: training confusion matrix (E), holdout validation confusion matrix (F), LV1 vs. LV2 score plot (G), and LV1 vs. LV3 score plot (H). Semi-dry vs. dry classification: training confusion matrix (I), holdout validation confusion matrix (J), LV1 vs. LV2 score plot (K), and LV1 vs. LV3 score plot (L). In the confusion matrices, the horizontal and vertical axes represent the observed and predicted classes, respectively; diagonal values indicate correct classifications, whereas off-diagonal values indicate misclassifications. In the score plots, samples are colored according to post-harvest processing method, and the ellipses illustrate the distribution of each class within the corresponding latent-variable projection.

**Fig. 6:**
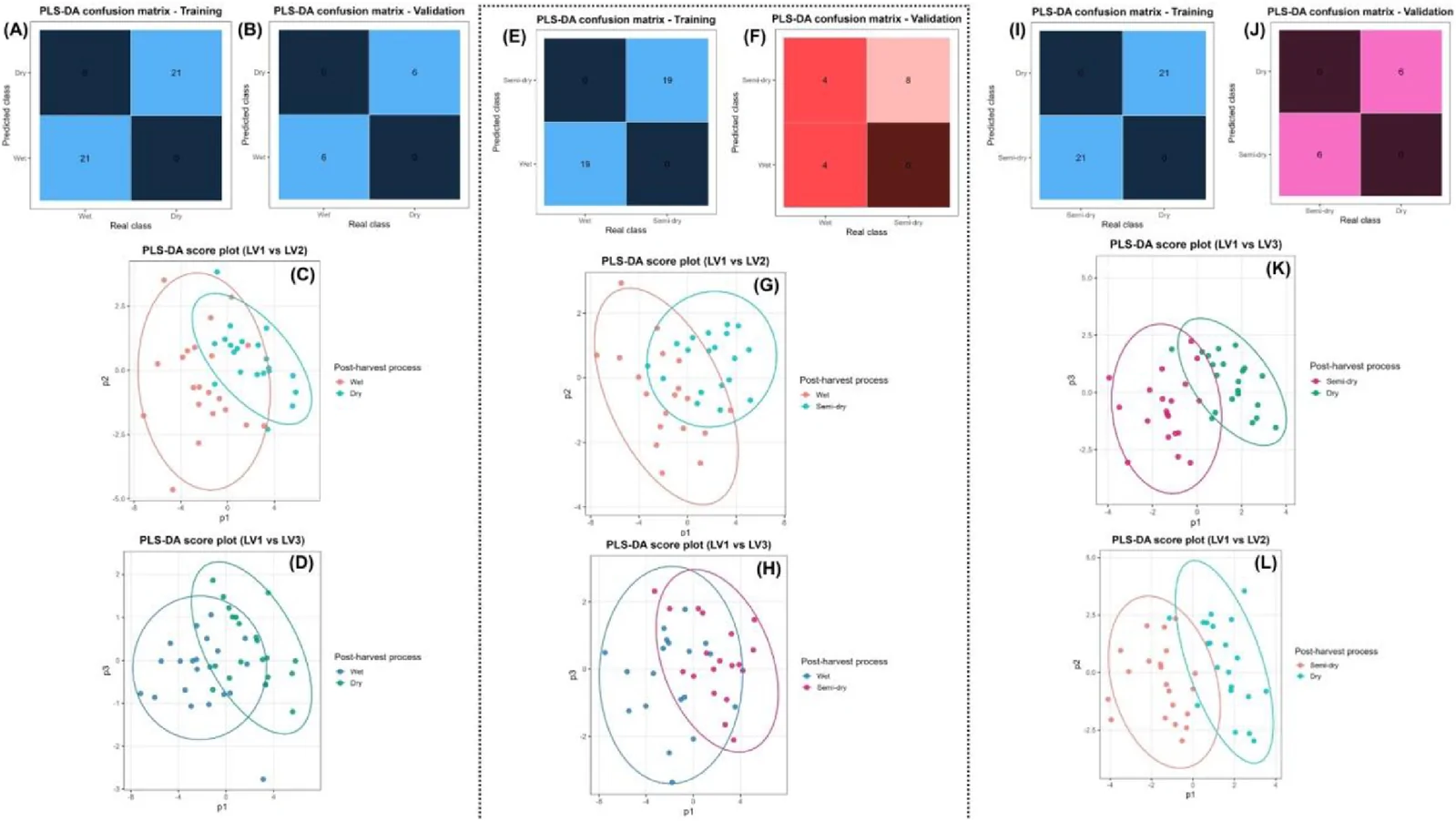
Classification performance and latent-variable score plots of Partial Least Squares Discriminant Analysis (PLS-DA) models developed using the features extracted from predefined spectral regions approach and roasted coffee samples for pairwise classification according to post-harvest processing method. Wet vs. dry classification: training confusion matrix (A), holdout validation confusion matrix (B), score plot of the first and second predictive latent variables (LV1 vs. LV2; C), and score plot of the first and third predictive latent variables (LV1 vs. LV3; D). Wet vs. semi-dry classification: training confusion matrix (E), holdout validation confusion matrix (F), LV1 vs. LV2 score plot (G), and LV1 vs. LV3 score plot (H). Semi-dry vs. dry classification: training confusion matrix (I), holdout validation confusion matrix (J), LV1 vs. LV2 score plot (K), and LV1 vs. LV3 score plot (L). In the confusion matrices, the horizontal and vertical axes represent the observed and predicted classes, respectively; diagonal values indicate correct classifications, whereas off-diagonal values indicate misclassifications. In the score plots, samples are colored according to post-harvest processing method, and the ellipses illustrate the distribution of each class within the corresponding latent-variable projection.

**Fig. 7:**
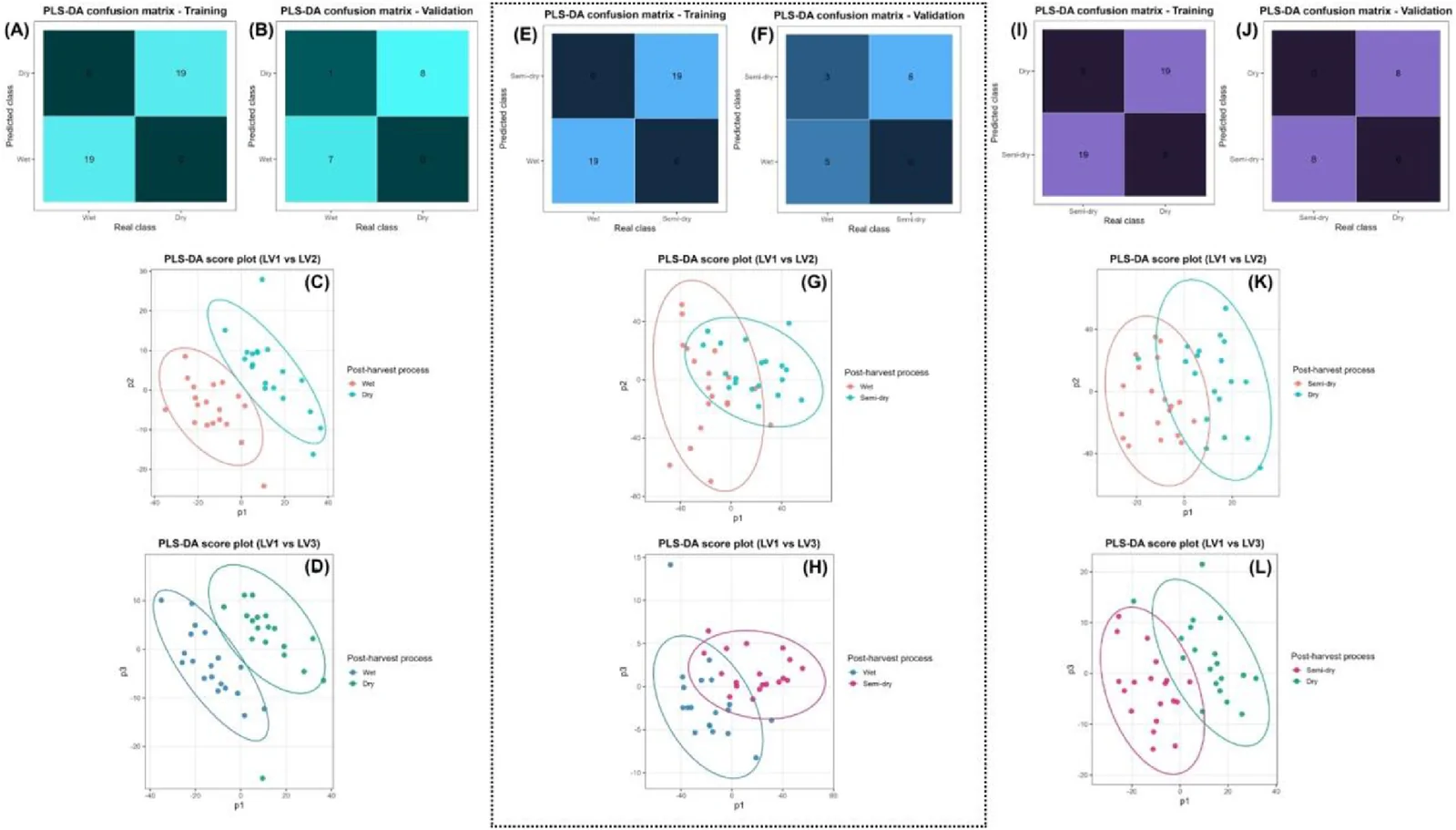
Classification performance and latent-variable score plots of Partial Least Squares Discriminant Analysis (PLS-DA) models developed using the features extracted from predefined spectral regions approach and green coffee samples for pairwise classification according to post-harvest processing method. Wet vs. dry classification: training confusion matrix (A), holdout validation confusion matrix (B), score plot of the first and second predictive latent variables (LV1 vs. LV2; C), and score plot of the first and third predictive latent variables (LV1 vs. LV3; D). Wet vs. semi-dry classification: training confusion matrix (E), holdout validation confusion matrix (F), LV1 vs. LV2 score plot (G), and LV1 vs. LV3 score plot (H). Semi-dry vs. dry classification: training confusion matrix (I), holdout validation confusion matrix (J), LV1 vs. LV2 score plot (K), and LV1 vs. LV3 score plot (L). In the confusion matrices, the horizontal and vertical axes represent the observed and predicted classes, respectively; diagonal values indicate correct classifications, whereas off-diagonal values indicate misclassifications. In the score plots, samples are colored according to post-harvest processing method, and the ellipses illustrate the distribution of each class within the corresponding latent-variable projection.

**Table 1:** Selected Partial Least Squares Discriminant Analysis models and classification performance results for discriminating coffee according to post-harvest processing method using mid-infrared spectra. Model performance is reported for the training set, K-out-of-fold cross-validation, and holdout validation using accuracy (A_cc_), Cohen’s kappa coefficient (CPC), sensitivity (S_e_), and specificity (S_p_).

Preprocessing	Modeling approach	Coffee stage	Classification	Comparison	Data partition	Training	Cross-Validation	External validation
D2	ii	Green	Multiclass	W vs. SD vs. Dr	75/25	A_cc_=1 CPC= 1 S_e_= 1 S_p_= 1	A_cc_=0.84 CPC= 0.76 S_e_= 0.84 S_p_= 0.92	A_cc_=1 CPC= 1 S_e_= 1 S_p_= 1
Raw	iv	Green	Multiclass	W vs. SD vs. Dr	70/30	A_cc_=0.94 CPC= 0.92 S_e_= 0.95 S_p_= 0.97	A_cc_=0.85 CPC= 0.78 S_e_= 0.86 S_p_= 0.93	A_cc_=0.83 CPC= 0.75 S_e_= 0.83 S_p_= 0.92
D2	i	Green	Binary	W vs. Dr	70/30	A_cc_=1 CPC= 1 S_e_= 1 S_p_= 1	A_cc_=0.84 CPC= 0.68 S_e_= 0.84 S_p_= 0.84	A_cc_=1 CPC= 1 S_e_= 1 S_p_= 1
Raw	i	Green	Binary	W vs. SD	70/30	A_cc_=1 CPC= 1 S_e_= 1 S_p_= 1	A_cc_=0.84 CPC= 0.68 S_e_= 0.84 S_p_= 0.84	A_cc_=0.94 CPC= 0.88 S_e_= 0.94 S_p_= 0.94
SNV	i	Green	Binary	SD vs. Dr	70/30	A_cc_=1 CPC= 1 S_e_= 1 S_p_= 1	A_cc_=0.89 CPC= 0.78 S_e_= 0.89 S_p_= 0.89	A_cc_=1 CPC= 1 S_e_= 1 S_p_= 1
SNV	ii	Roasted	Binary	W vs. Dr	70/30	A_cc_=1 CPC= 1 S_e_= 1 S_p_= 1	A_cc_=1 CPC= 1 S_e_= 1 S_p_= 1	A_cc_=1 CPC= 1 S_e_= 1 S_p_= 1
Raw	ii	Roasted	Binary	W vs. SD	70/30	A_cc_=1 CPC= 1 S_e_= 1 S_p_= 1	A_cc_=0.82 CPC= 0.63 S_e_= 0.82 S_p_= 0.82	A_cc_=0.94 CPC= 0.89 S_e_= 0.94 S_p_= 0.94
D2	ii	Roasted	Binary	SD vs. Dr	75/25	A_cc_=1 CPC= 1 S_e_= 1 S_p_= 1	A_cc_=0.81 CPC= 0.62 S_e_= 0.81 S_p_= 0.81	A_cc_=0.92 CPC= 0.83 S_e_= 0.92 S_p_= 0.92
MSC	iv	Green	Binary	W vs. Dr	75/25	A_cc_=1 CPC= 1 S_e_= 1 S_p_= 1	A_cc_=0.88 CPC= 0.76 S_e_= 0.88 S_p_= 0.88	A_cc_=1 CPC= 1 S_e_= 1 S_p_= 1
D1	iv	Green	Binary	W vs. SD	70/30	A_cc_=1 CPC= 1 S_e_= 1 S_p_= 1	A_cc_=0.79 CPC= 0.58 S_e_= 0.79 S_p_= 0.79	A_cc_=0.75 CPC= 0.75 S_e_= 0.75 S_p_= 0.75
SNV	iv	Green	Binary	SD vs. Dr	75/25	A_cc_=1 CPC= 1 S_e_= 1 S_p_= 1	A_cc_=0.93 CPC= 0.86 S_e_= 0.93 S_p_= 0.93	A_cc_=1 CPC= 1 S_e_= 1 S_p_= 1

Modeling approaches correspond to: (i) full-spectrum analysis, (ii) full-spectrum analysis restricted to selected spectral regions, (iii) global feature-extracted representation, and (iv) feature extraction within selected spectral regions.

The illustrative results presented in Fig. 3, Fig. 4, Fig. 5, Fig. 6, Fig. 7 demonstrate the suitability of the proposed **PLSDA_Modeling_Pipelines_Advanced.R** framework for discriminating coffee samples according to post-harvest processing under both multiclass and binary classification scenarios. The confusion matrices showed a high proportion of correctly classified samples in both the training and validation datasets, indicating that the PLS-DA models were able to capture relevant multivariate spectral information associated with W, SD, and Dr processing.

The latent-variable score plots (Figs. 3 to 7) complement these classification results by visualizing the multivariate structure extracted by the models. The first three predictive latent variables captured relevant discriminant information associated with post-harvest processing, with the LV1 vs. LV2 and LV1 vs. LV3 projections providing complementary views of the separation among W, SD, and Dr samples. Although the degree of separation varied depending on the processing comparison and modeling configuration, the distribution of the samples in these latent-variable spaces revealed systematic class-related patterns consistent with the high classification performance observed in the training and holdout validation sets (Table 1). Importantly, the information represented by LV2 and LV3 was complementary rather than redundant, indicating that discrimination among processing methods was distributed across multiple latent dimensions. Therefore, interpretation of the PLS-DA models should not rely exclusively on the LV1 vs. LV2 projection, as the inclusion of LV3 may reveal additional discriminant structure that is less evident in the first two-dimensional representation. Taken together, the score plots, confusion matrices, and classification metrics indicate that PLS-DA effectively captured the multivariate spectral differences associated with the different post-harvest processing methods within the evaluated coffee samples.

The statistical classification results summarized in Table 1 show consistently high performance across several modeling configurations, with some models achieving complete classification in both the training and holdout validation sets, while cross-validation accuracies generally remained high. These findings support the suitability of the PLS-DA approach for extracting discriminant information associated with coffee post-harvest processing and demonstrate that the proposed pipeline is capable of identifying modeling configurations with strong and consistent predictive performance across training, cross-validation, and holdout assessment.

The cases presented in Fig. 3, Fig. 4, Fig. 5, Fig. 6, Fig. 7 and Table 1 represent only a subset of the analytical possibilities enabled by the proposed framework. The workflow allows the systematic exploration of six spectral preprocessing strategies (raw, baseline correction, SNV, MSC, D1, and D2), four modeling approaches (i, ii, iii, and iv), three coffee-stage configurations (green, roasted, and green and roasted samples analyzed together), four classification scenarios (W vs. SD vs. Dr, W vs. SD, W vs. Dr, and SD vs. Dr), and three training/holdout partitions (70%/30%, 75%/25%, and 80%/20%), resulting in up to 864 possible analytical configurations. This flexibility enables the evaluation of both multiclass discrimination among W, SD, and Dr samples and the three corresponding pairwise binary classification problems, while simultaneously assessing the influence of preprocessing strategy, predictor representation, coffee stage, and data partition on model performance. Consequently, the framework provides a comprehensive and reproducible environment for comparing alternative PLS-DA configurations and identifying those most suitable for a given classification objective.

In the case of models developed using green and roasted coffee samples simultaneously (Fig. 8 and Table 2), this strategy may provide a broader representation of the spectral variability present across coffee processing stages. However, its interpretation requires particular attention because roasting induces pronounced physicochemical and spectral modifications (Fig. 1) that may contribute strongly to the latent structure extracted by the PLS-DA model. Consequently, when green and roasted samples are modeled together, part of the variance captured by the predictive latent variables may be associated with coffee stage itself (Fig. 8A), in addition to the spectral differences related to post-harvest processing method. This effect may reduce the relative contribution of the subtler discriminant information associated specifically with W, SD, and Dr processing (Fig. 8B). For this reason, the proposed framework allows the calibration of both stage-specific models, considering green or roasted coffee independently, and combined models incorporating both stages. This flexibility enables their relative performance to be evaluated empirically and allows the user to determine whether a broader combined model or more specific stage-dependent models are more appropriate for the classification objective under study.

**Fig. 8:**
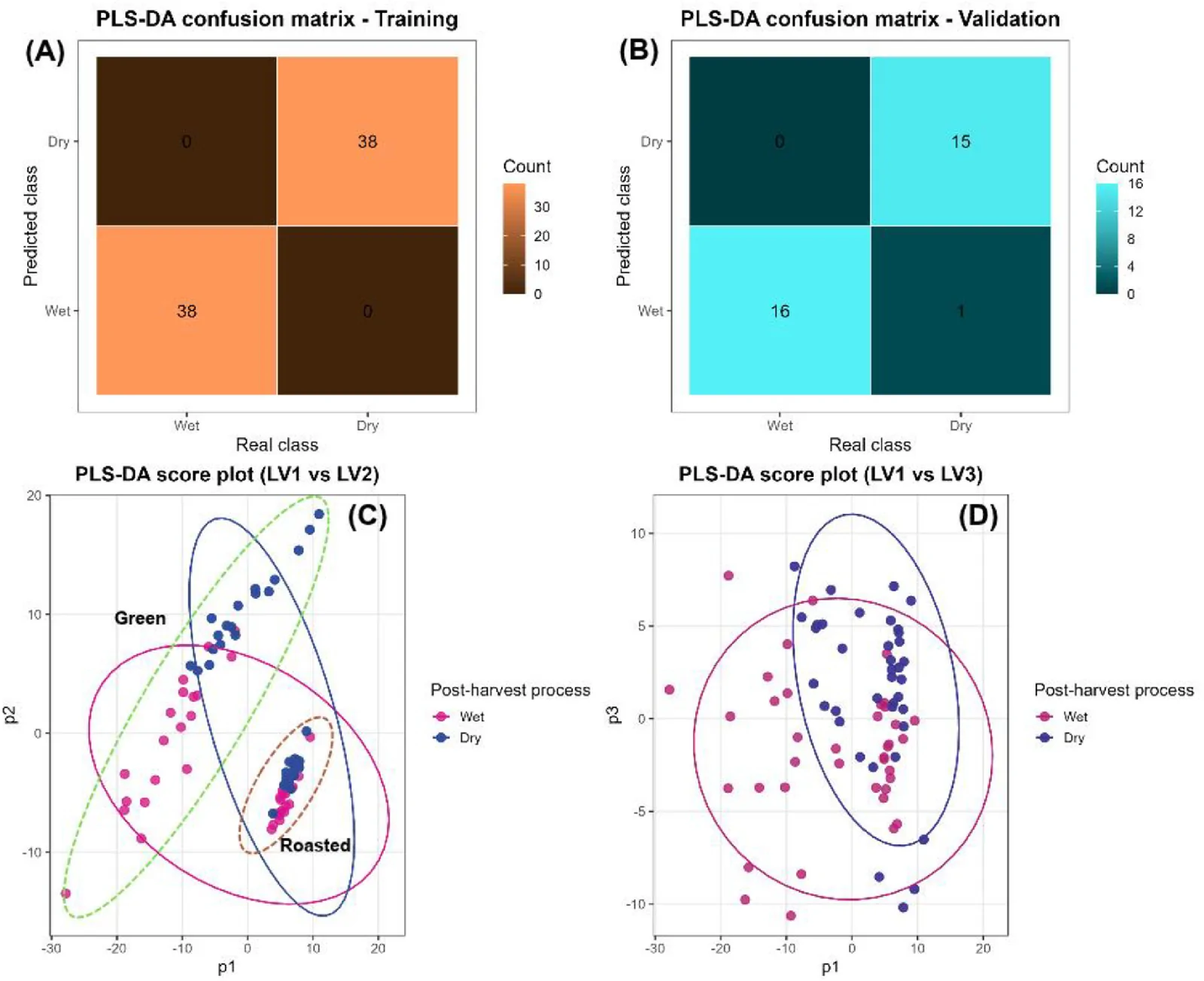
Classification performance and latent-variable score plots of the Partial Least Squares Discriminant Analysis (PLS-DA) model developed using the full-spectrum data restricted to predefined spectral regions and green/roasted coffee samples for classification according to post-harvest processing method (wet vs. dry). Training confusion matrix (A) and holdout validation confusion matrix (B), showing the relationship between observed (horizontal axis) and predicted (vertical axis) classes. Diagonal values represent correctly classified samples, whereas off-diagonal values indicate misclassifications. Score plot of the first and second predictive latent variables (LV1 vs. LV2; C) and score plot of the first and third predictive latent variables (LV1 vs. LV3; D). Samples are colored according to post-harvest processing method, and the ellipses illustrate the distribution of each class within the corresponding latent-variable projection.

**Table 2:** Statistical classification performance of the Partial Least Squares Discriminant Analysis (PLS-DA) model developed to discriminate wet and dry post-harvest processing methods using full-spectrum data restricted to predefined spectral regions and considering green and roasted coffee samples simultaneously. Model performance is reported for the training set, K-fold out-of-fold cross-validation, and holdout validation using accuracy (A_cc_), Cohen’s kappa coefficient (CPC), sensitivity (S_e_), and specificity (S_p_).

Preprocessing	Modeling approach	Coffee stage	Classification	Comparison	Data partition	Training	Cross-Validation	External validation
D1	ii	Green and roasted	Binary	W vs. Dr	70/30	A_cc_=1 CPC= 1 S_e_= 1 S_p_= 1	A_cc_=0.95 CPC= 0.89 S_e_= 0.95 S_p_= 0.95	A_cc_=1 CPC= 1 S_e_= 1 S_p_= 1

Modeling approach (ii) is full-spectrum analysis restricted to selected spectral regions.

The **PLSDA_Wavelet_DB4_Modeling.R** script extends the previously described PLS-DA workflow to the analysis of wavelet-derived spectral features. It directly imports the precomputed Daubechies wavelet of order four (db4) datasets by **FeatureExtraction_SpecificSpectralRegionWavelet.m** and provided in **WaveletSpectralAnalysis.xlsx**, allowing users to select among raw, baseline-corrected, SNV, MSC, D1, and D2 spectral preprocessing strategies. Each dataset comprises 18 wavelet-derived features, obtained by calculating INT, ENG, STD, SKE, KURT, and ENT independently within three specific spectral regions. The script supports multiclass and pairwise binary discrimination of wet, semi-dry, and dry post-harvest processing methods using green coffee, roasted coffee, or both stages. Following the validation and reporting procedures established in the main pipeline, it provides training, five-fold out-of-fold cross-validation, and holdout validation results, together with classification metrics, confusion matrices, and graphical diagnostics. This complementary workflow enables the investigation of the discriminatory information modeled in wavelet-transformed spectral features and their potential contribution to coffee post-harvest processing classification.

An example of the application of this complementary workflow is presented in Fig. 9 for the binary discrimination of W and Dr green coffee samples using MSC-preprocessed spectra and the 18 regional db4 wavelet-derived features. The selected model showed high classification performance with training accuracy of 0.97, five-fold out-of-fold cross-validation accuracy of 0.79, and holdout validation accuracy of 0.94. Cohen’s kappa coefficients were 0.95, 0.58, and 0.88 for training, cross-validation, and holdout validation, respectively, while sensitivity and specificity followed comparable trends (>0.92 for training and validation). These results indicate that the wavelet-derived representation retained relevant discriminatory information associated with the post-harvest processing method. Additionally, the latent-variable score plots provide a complementary visualization of this multivariate structure, showing differentiation between W vs. Dr processed samples in the LV1-LV2 and LV1-LV3 projections. Finally, this example illustrates how the wavelet-based workflow can be used to investigate whether wavelet transformation enhances the representation of relevant spectral information and, consequently, improves the discrimination of coffee samples according to post-harvest processing.

**Fig. 9:**
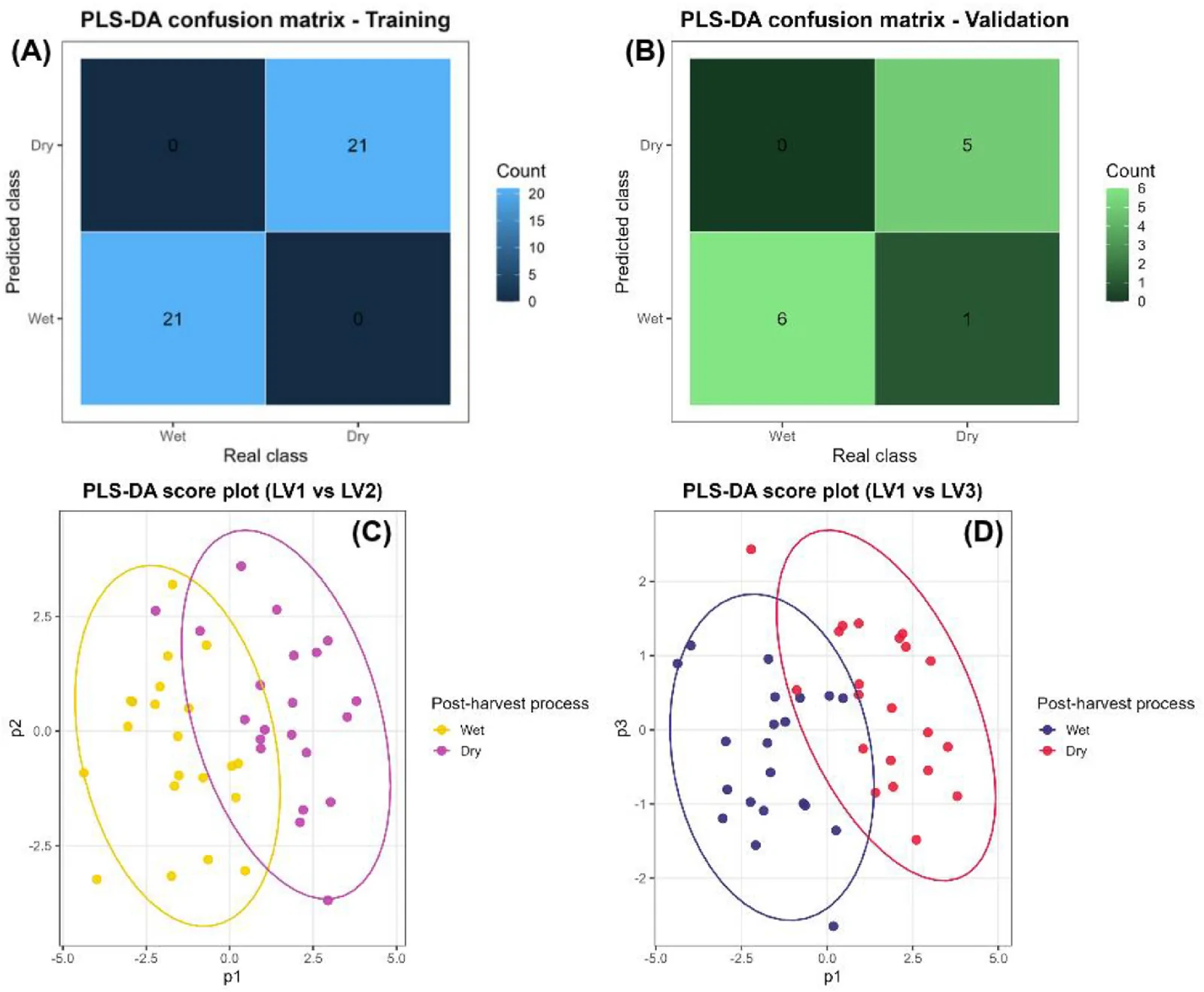
Classification performance and latent-variable score plots of the Partial Least Squares Discriminant Analysis (PLS-DA) model developed using the Daubechies wavelet of order four (db4) wavelet-derived features from Multiplicative Scatter Correction-preprocessed spectra of green coffee samples for binary discrimination between wet and dry post-harvest processing methods. Training confusion matrix (A) and holdout validation confusion matrix (B), showing the relationship between observed (horizontal axis) and predicted (vertical axis) classes. Correct and misclassified samples are identified from the corresponding observed-predicted class combinations. Score plot of the first and second predictive latent variables (LV1 vs. LV2; C) and score plot of the first and third predictive latent variables (LV1 vs. LV3; D).

An additional documentation file, **README_PLSDA_Wavelet_DB4**, is provided to guide users in the appropriate implementation of the accompanying computational tools. It outlines recommended practices for data preparation, model configuration, validation, and interpretation of PLS-DA results, thereby promoting reproducible and methodologically consistent chemometric analyses.

The potential applicability of the dataset and its associated modeling framework extends beyond the examples presented and can be further explored through statistical diagnostics aimed at determining whether new observations fall within the calibration domain of a previously developed model. Both **PLSDA_Modeling_Pipelines_Advanced.R** and **PLSDA_Wavelet_DB4_Modeling.R** allow the calibrated PLS-DA model to be saved as an RDS object, enabling its subsequent application to independent sets of mid-infrared spectra. To support this future use, the framework incorporates two complementary multivariate statistical diagnostics: the squared prediction error (SPE), which quantifies the residual variation of a new observation in the X-space, and Hotelling’s T-squared (T^2^), which evaluates its position within the latent-variable space defined by the calibration model [12]. Together, these statistics provide an applicability-domain assessment that can be used to identify incoming observations that are inconsistent with the multivariate correlation structure learned during calibration. Samples exceeding the corresponding control limits can therefore be flagged as atypical or out-of-domain observations, and their classification should be interpreted with caution rather than being automatically accepted as reliable model predictions. As an illustrative example, a PLS-DA model was calibrated exclusively using roasted coffee spectra and subsequently challenged with green coffee samples that were not included during model calibration. As shown in Fig. 10, the green coffee samples exceeded the 99% control limits for both SPE and T^2^, whereas the roasted coffee samples remained within the corresponding limits. This result demonstrates that the statistical diagnostics were able to identify samples presenting a spectral structure substantially different from that represented in the calibration space.

**Fig. 10:**
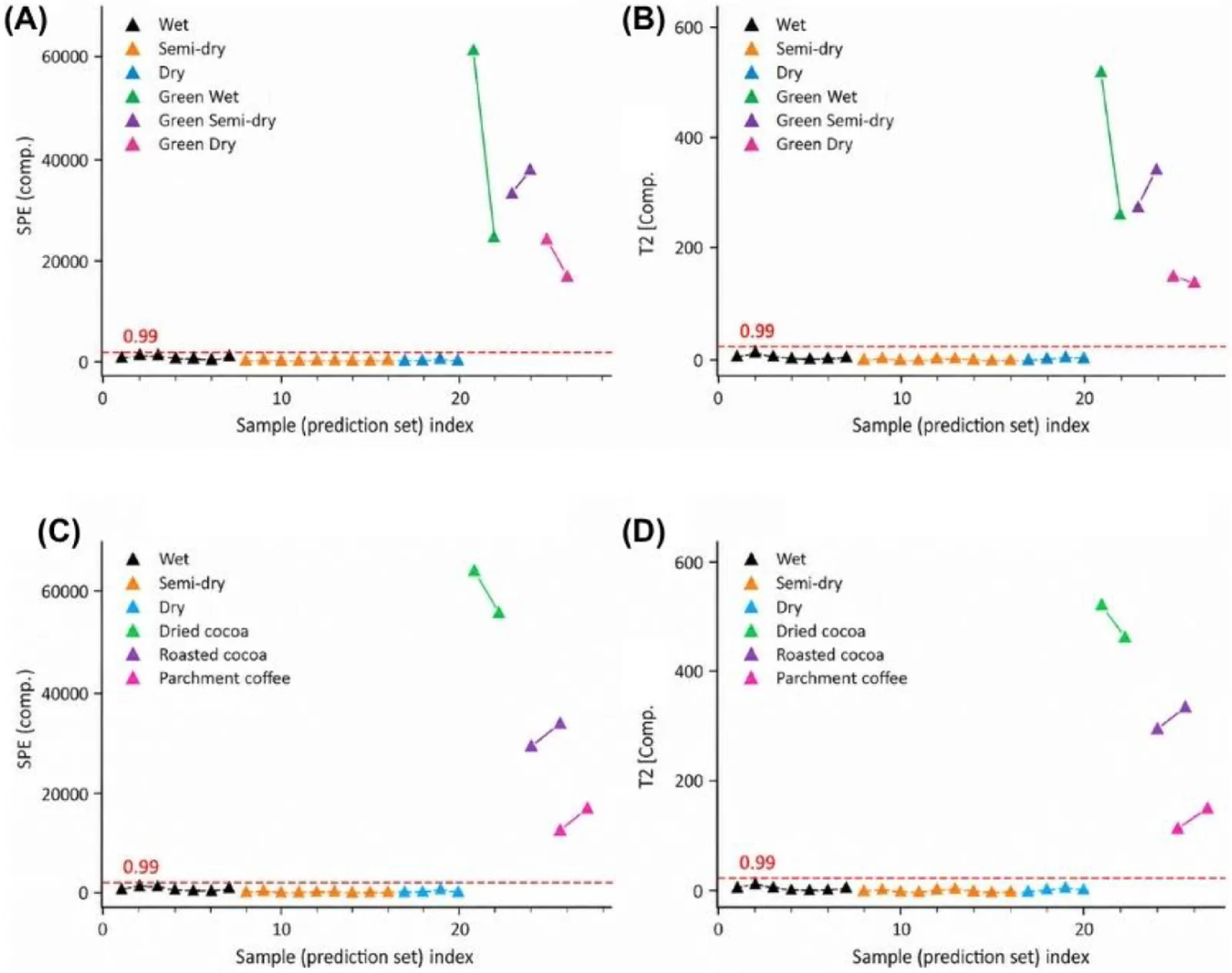
Diagnostic assessment of a Partial Least Squares Discriminant Analysis model calibrated using roasted coffee spectra, based on squared prediction error (SPE) and Hotelling’s T-squared (T^2^). Panels A to B illustrate the evaluation of incoming green coffee spectra, whereas panels C to D extend the assessment to dried cocoa, roasted cocoa, and parchment coffee spectra. The red dashed line indicates the 99% control limit. In both examples, the incoming samples exceed the corresponding limits, indicating that their spectral profiles fall outside the multivariate calibration space defined by the roasted coffee model.

A further illustrative example involving different coffee matrices and related food products is presented in Fig. 10, Fig. 10. In this case, mid-infrared spectra of dried cocoa, roasted cocoa, and parchment coffee reported in previous studies [23,24] were introduced as external observations into the calibrated PLS-DA model. As shown in Fig. 10C, these samples exhibited markedly higher SPE values than the samples belonging to the original calibration domain and exceeded the corresponding 99% control limit. A similar behavior was observed for T^2^ (Fig. 10D), where dried cocoa, roasted cocoa, and parchment coffee were clearly positioned above the statistical control limit. These results indicate that the multivariate statistical diagnostics were able to identify samples whose spectral structure differed substantially from that represented by the calibration dataset. Thus, the combined use of SPE and T^2^ provides a practical mechanism for detecting incoming spectra that fall outside the model applicability domain before their classification results are interpreted. Importantly, this example should not be considered an external validation of the coffee-processing classification model, since these materials were intentionally selected as spectrally different challenge samples. Rather, it illustrates how the proposed framework can support future applications by identifying observations for which model extrapolation would be inappropriate and for which model expansion or recalibration may be required.

## 4 Experimental Design, Materials and Methods

Eighty coffee cherries samples (20 kg each; Fig. 11) of the Bourbon Rosado variety (*Coffea arabica* L.) were harvested directly from coffee farms located in the San-Adolfo in Huila region of Colombia. All samples were obtained from growing areas and transported to the Centro Surcolombiano de Investigación en Café (CESURCAFÉ), Universidad Surcolombiana, Neiva, Huila, Colombia. During transport, the samples were kept under refrigerated conditions in coolers for approximately 5 h at temperatures below 5°C to minimize spontaneous fermentation before processing [25]. Once coffee samples arrived at CESURCAFÉ, the coffee cherries were washed and immersed in water to remove floating, immature, or defective fruits. Then, the coffee cherry beans were divided into three processing batches (27 samples in each bath) corresponding to wet processing (W), semi-dry processing (SD) and Dry processing (Dr). In the case of W and SD, the selected cherries were mechanically depulped using a coffee pulping machine (Gaviota GV 300, INGESEC, Colombia) [25]. For W processing, the depulped coffee beans were fermented in plastic containers during 18 h [26]. After fermentation, W coffee beans were washed again to remove the remaining mucilage and subsequently sun-dried. For SD processing, depulped coffee beans were immediately subjected to sun drying without an intermediate fermentation step. In the case of Dr processing, whole coffee cherries were directly sun-dried without depulping (Fig. 11). The sun-drying process consisted of reducing the moisture content of the beans through direct exposure to solar energy under natural environmental conditions. During this stage, the samples were spread in thin layers to promote uniform dehydration and were periodically mixed to avoid localized moisture accumulation and ensure homogeneous drying. This operation was continued until the beans reached the moisture level of 10-12% on a wet basis, required for safe storage and subsequent processing. Moisture content was monitored during drying using a portable grain moisture meter (Kett PM-450, Science of Sensing, Japan). Sun-drying was performed daily from 9:00 a.m. to 4:00 p.m. under environmental conditions of 30-43°C and 25-53% relative humidity (Fig. 12). Once the target moisture content was reached, the dried parchment coffee (Fig. 11) was hulled using a mechanical hulling machine (ING-C-250, INGESEC, Colombia; Fig. 13) to obtain green coffee beans. Green coffee of W, SD and Dr were roasted using a rotary roasting system (Prisma TECH DR150g, Prisma Coffee Tech, Colombia), which allowed controlled adjustment of roasting time and temperature. Before roasting, the system was preheated at 155°C for 10 min to stabilize the roasting chamber. The chamber temperature was then increased to 170 ± 3°C and allowed to stabilize at 171 ± 3°C before loading the green coffee beans [27]. During roasting, bean temperature was continuously recorded at 5 s intervals. After 4 min of roasting, the heating power was increased from 15% to 65% to promote roasting development (Fig. 14). The roasting process was completed after 11 ± 0.5 min, when the beans reached a final temperature of 182 ± 2°C. The roasted samples were subsequently cooled under ambient conditions. Green and roasted coffee beans from W, SD and Dr were initially characterized by determining their moisture content (MC; % wet basis, %w.b.), water activity (a_w_) and color (Tables 3 and 4). For MC, 10 g of coffee sample was placed in an oven (UF55, Memmert GmbH + Co. KG, Schwabach, Germany) at 105°C for approximately 24 h until the weight of the coffee beans was constant. The a_w_ was determined using a vapor sorption analyzer (VSA Aqualab Decagon Devices, Inc. Pullman, WA). In every trial, 5.0 g of coffee sample was used, and the analysis was conducted in triplicate for both moisture content and a_w_. Furthermore, color properties of coffee samples were quantified via the CieLab color system (L: lightness, a: redness, and b: greenness) using a spectrocolorimeter (CR-410, Konica Minolta, NJ, USA), considering standard light source D65 and standard observer 10°. A standard white plate was used to calibrate the instrument (Y = 87.0, x = 0.3160, y = 0.3231) [28].

**Fig. 11:**
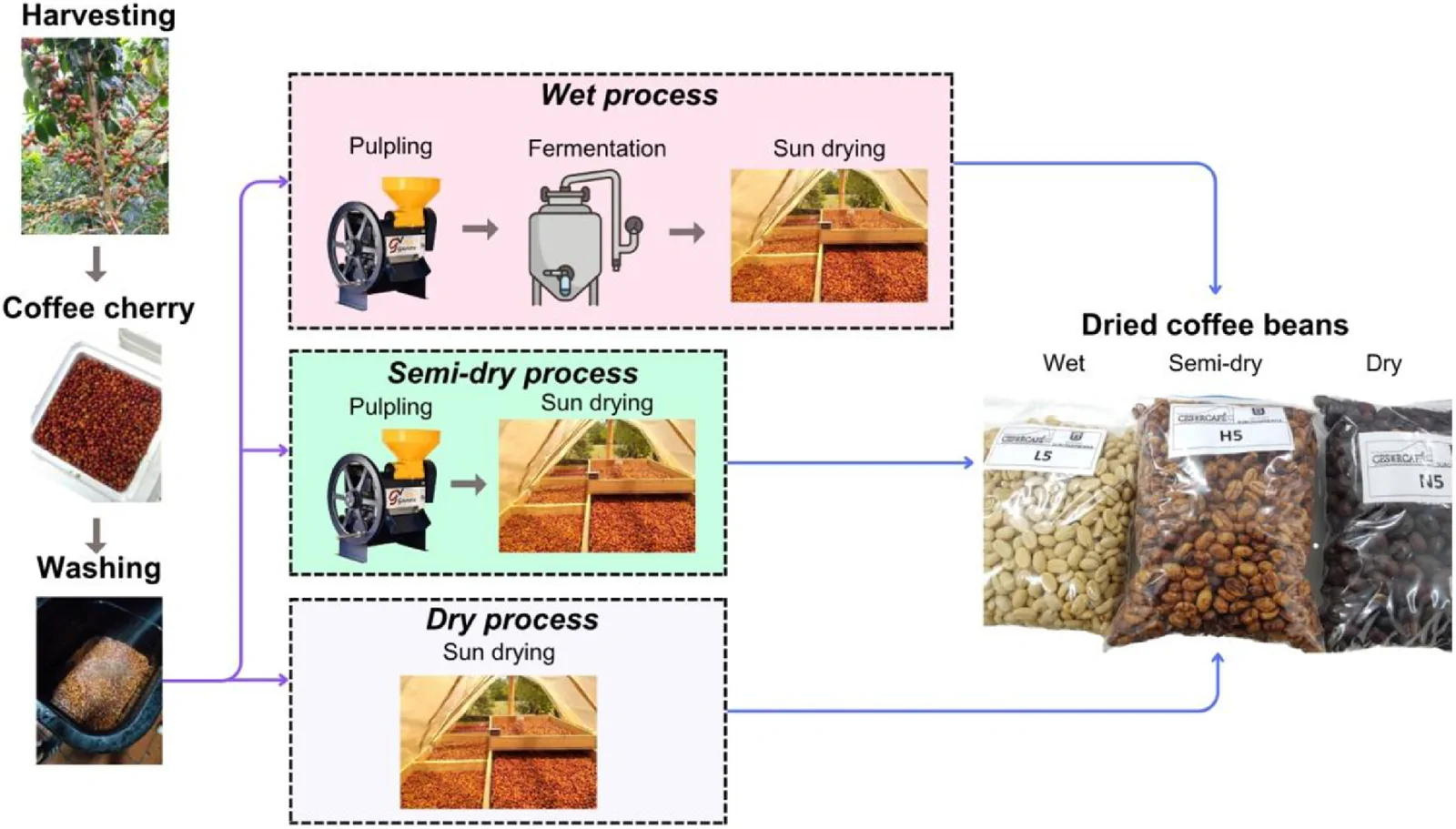
Post-harvest processing workflow used to obtain dried specialty coffee beans according to wet, semi-dry and dry processing methods. Coffee cherries were harvested and initially washed before being subjected to three different post-harvest methods. In the wet process, cherries underwent pulping, fermentation and sun drying. In the semi-dry process, cherries were pulped and subsequently sun-dried without a fermentation step. In the dry process, whole coffee cherries were directly sun-dried. The resulting dried coffee beans from the wet, semi-dry and dry processes were used for subsequent spectral analysis and mathematical modeling.

**Fig. 12:**
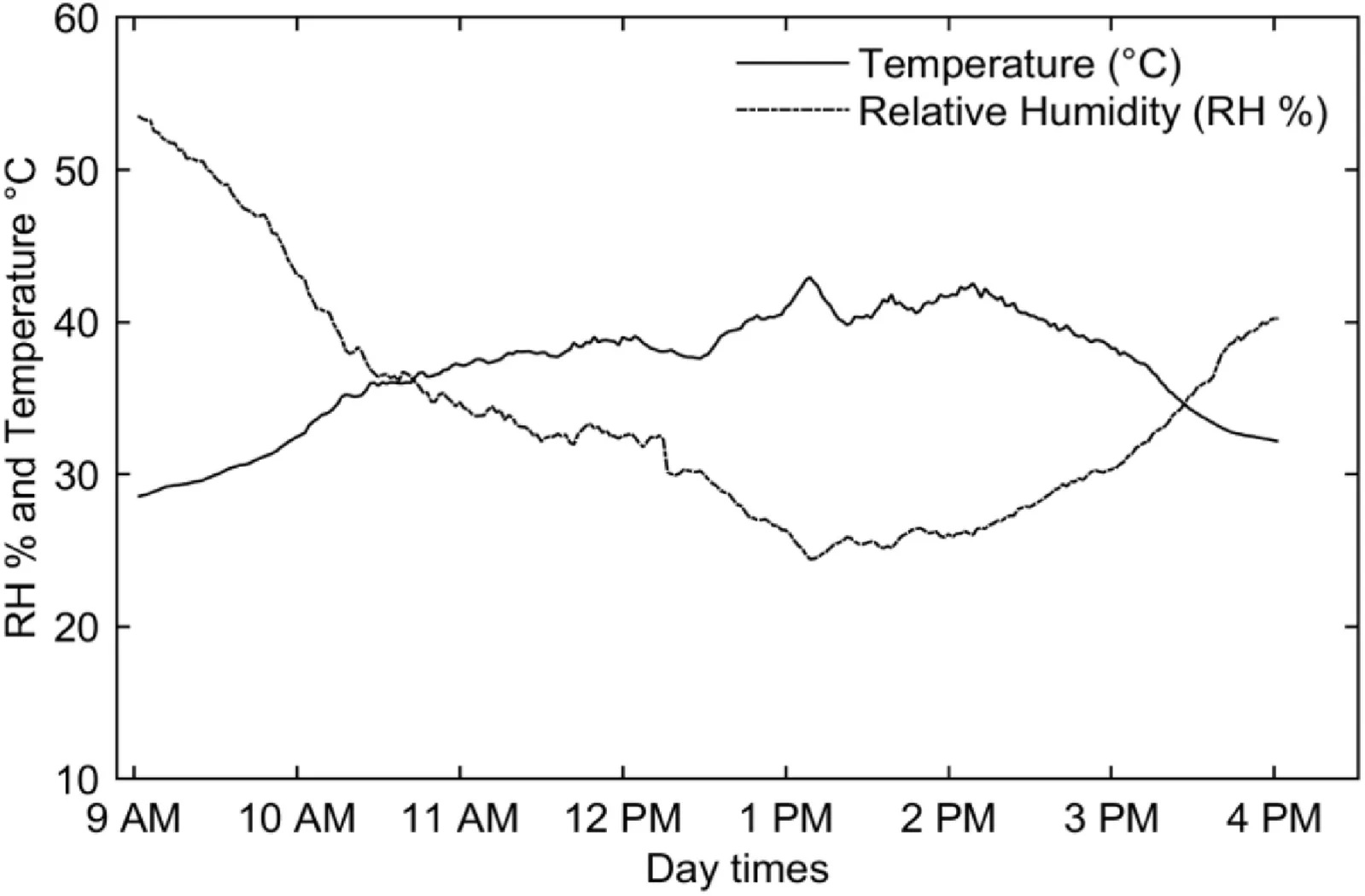
Variation in temperature and relative humidity during the sun-drying of coffee. The solid line represents temperature, while the dashed line represents relative humidity recorded throughout the drying period.

**Fig. 13:**
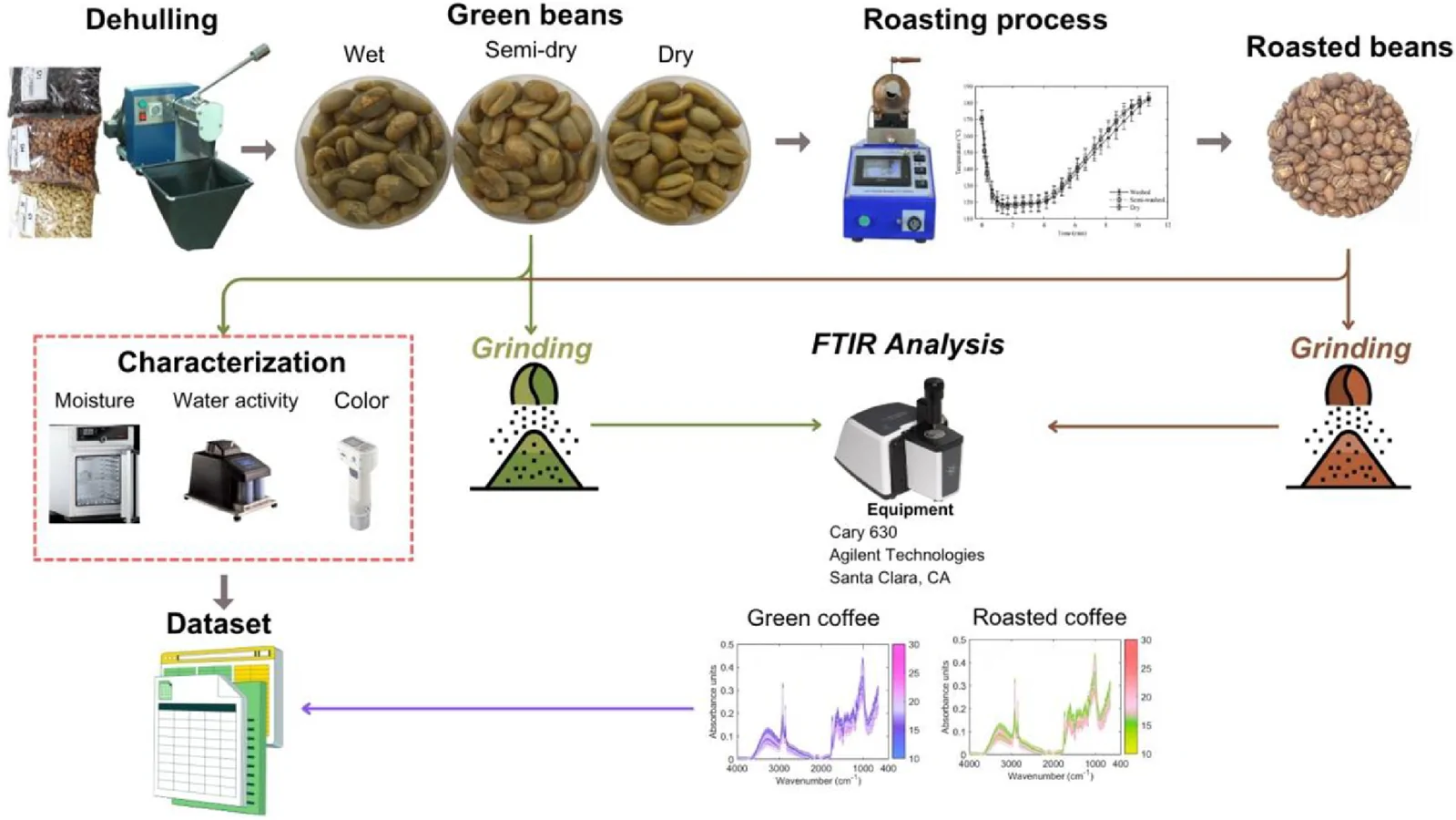
Experimental workflow for the development of the mid-infrared spectral dataset of green and roasted specialty coffee. Dried coffee beans obtained from wet, semi-dry and dry post-harvest processing methods were dehulled to obtain green coffee beans. The green beans were initially characterized through moisture content, water activity and color measurements. Subsequently, part of the green coffee samples was ground for Fourier transform mid-infrared spectral acquisition, while another portion was subjected to roasting under controlled conditions. The roasted beans were also ground and analyzed by Fourier transform mid-infrared spectroscopy. The spectral information obtained from green and roasted coffee samples was used to construct the dataset for subsequent preprocessing, MATLAB-based feature extraction and R-based Partial Least Squares Discriminant Analysis modeling.

**Fig. 14:**
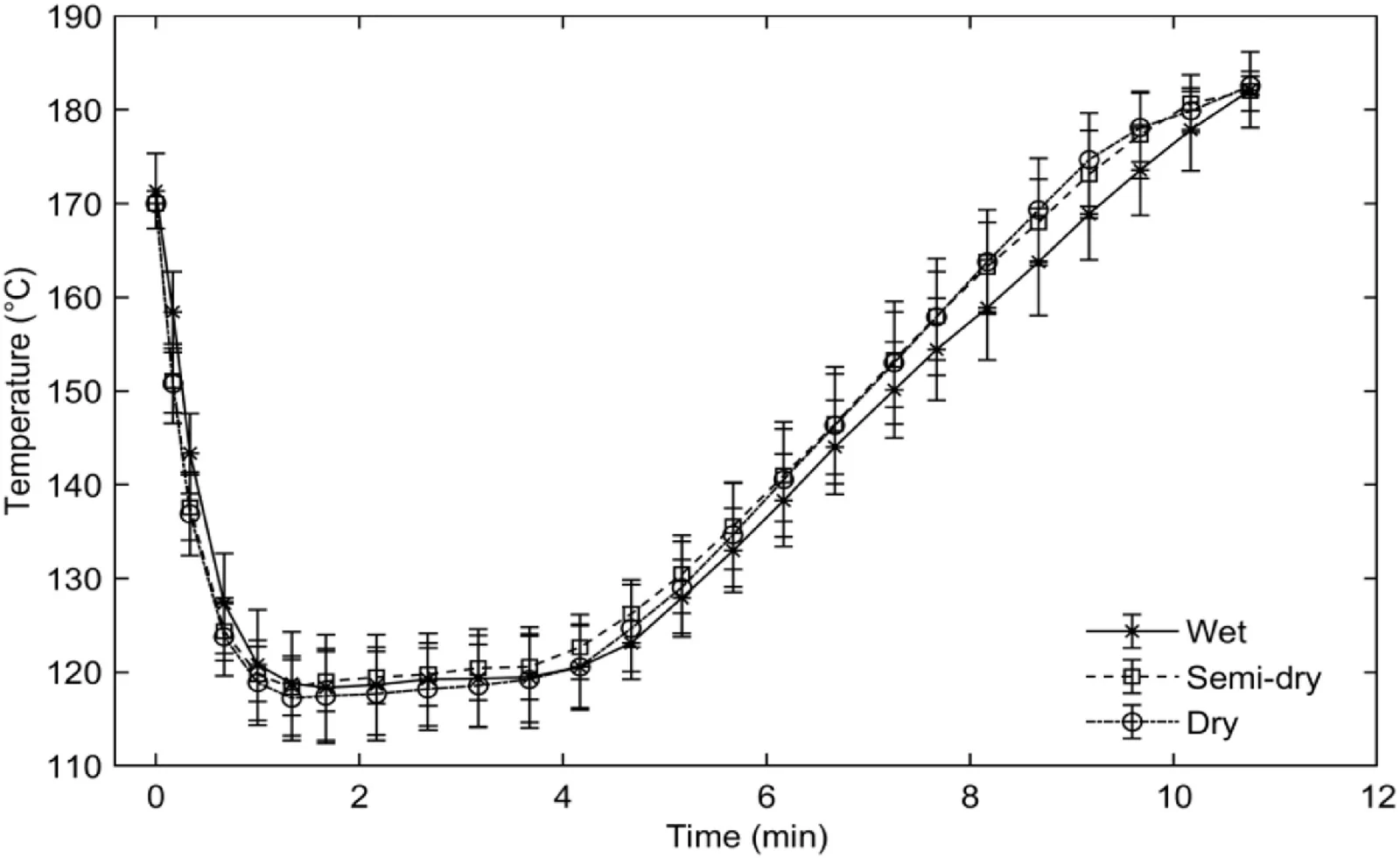
Temperature profiles recorded during the roasting of specialty coffee beans obtained from wet, semi-dry and dry post-harvest processing methods. The curves show the evolution of roasting temperature as a function of time for each processing method, with error bars representing experimental variability. All samples exhibited an initial temperature decrease followed by a progressive increase until reaching final roasting temperatures close to 180°C. The profiles were used to characterize the thermal conditions applied during roasting before mid-infrared spectral acquisition and subsequent mathematical modeling.

**Table 3:** Initial characterization of green coffee samples processed by different post-harvest methods. Results are expressed as mean ± standard deviation.

Green coffee					
Processing	MC%	a_w_	L*	a*	b*
Wet	10.94±0.67	0.56±0.03	39.39±0.77	1.07±0.13	9.39±0.33
Semi-dry	11.11±0.20	0.58±0.01	38.76±1.76	2.02± 0.30	10.62±0.67
Dry	10.13±0.26	0.55±0.01	40.48±0.95	1.83± 0.13	12.01± 0.62

**Table 4:** Initial characterization of roasted coffee samples processed by different post-harvest methods. Results are expressed as mean ± standard deviation.

roasted coffee					
Processing	MC%	a_w_	L*	a*	b*
Wet	2.08± 0.38	0.30± 0.02	23.53± 0.56	4.56± 0.23	5.10± 0.38
Semi-dry	2.09± 0.16	0.31± 0.02	23.29± 0.62	4.80± 0.28	5.13± 0.36
Dry	2.16± 0.25	0.34± 0.03	23.35± 0.46	5.01± 0.23	5.24± 0.38

Green coffee samples showed moisture content values between 10.13 ± 0.26% and 11.11 ± 0.20%, while a_w_ ranged from 0.55 ± 0.01 to 0.58 ± 0.01. Regarding color parameters, green coffee samples exhibited L* values from 38.76 ± 1.76 to 40.48 ± 0.95, a* values from 1.07 ± 0.13 to 2.02 ± 0.30, and b* values from 9.39 ± 0.33 to 12.01 ± 0.62. After roasting, moisture content decreased markedly, with values ranging from 2.08 ± 0.38% to 2.16 ± 0.25%, confirming the intense water loss produced during thermal processing. a_w_ also decreased substantially, reaching values between 0.30 ± 0.02 and 0.34 ± 0.03, indicating reduced water availability in roasted coffee samples. Roasting also produced clear changes in color parameters. The L* values decreased to approximately 23.29-23.53, reflecting the darkening of coffee beans during roasting. In contrast, a* values increased to 4.56-5.01, indicating a shift toward redder tones, while b* values decreased to 5.10-5.24, suggesting a reduction in yellow coloration compared with green coffee.

To determine whether the coffee samples processed by W, SD and Dr post-harvest methods could be classified as specialty coffee, sensory quality assessment was performed according to the Specialty Coffee Association cupping protocol (SCA, 2020). The evaluation was conducted at CESURCAFÉ by five highly trained coffee sensory panelists, following the criteria described by [29]. The evaluated attributes included fragrance, flavor, aftertaste, acidity, body, taster score and overall cup quality score (Table 5). As shown, all coffee samples obtained overall scores above 80 points, confirming their classification as specialty coffee according to the SCA quality scale [30]. W-processed coffee showed the highest overall score (83.55 ± 1.00), followed by SD (83.14 ± 1.34) and Dr-processed coffee (82.43 ± 0.94). A similar trend was observed for most sensory attributes, with W, SD and Dr samples presenting slightly higher fragrance, flavor, aftertaste, acidity, body and taster scores than DR-processed samples. These results indicate that all post-harvest processing methods produced specialty-grade coffee, while slight differences in sensory profiles may reflect the influence of processing conditions on the final cup quality.

**Table 5:** Sensory quality attributes of roasted specialty coffee samples obtained from wet, semi-dry and dry post-harvest processing methods. Results are expressed as mean ± standard deviation.

Processing	Fragrance	Flavor	Aftertaste	Acidity	Body	Taster Score	Overall score
Wet	8.20±0.09	7.68±0.14	7.66±0.15	7.31±0.14	7.42±0.18	6.91±0.19	83.55±1.00
Semi-dry	8.21±0.15	7.64±0.12	7.57±0.18	7.29±0.13	7.35±0.23	6.86±0.26	83.14±1.34
Dry	8.14±0.12	7.50±0.18	7.47±0.19	7.20±0.16	7.30±0.20	6.68±0.11	82.43±0.94

Before spectral acquisition, green and roasted specialty coffee samples obtained from W, SD and Dr post-harvest processing methods were ground to obtain a homogeneous particle-size distribution (Fig. 13). Due to the higher mechanical resistance of green coffee beans, a two-stage grinding procedure was applied. First, green beans were coarsely ground using a rotary blade grinder (FPSTFP3322, Oster®, Colombia) to reduce particle size. The resulting material was then finely milled using a burr grinder (G3 HD BLK, Bunn®, Springfield, USA). In contrast, roasted coffee beans were directly ground using the same burr grinder because of their greater brittleness. The resulting powders were sieved using a vibratory sieve shaker (EFL-2000, Endecotts Ltd., London, UK) for 20 min to obtain a controlled particle-size distribution. The fraction retained on a #60 sieve (250 µm aperture) was selected to ensure sample homogeneity, whereas finer particles were discarded due to their negligible proportion. Ground samples were subsequently stored in airtight containers until analysis to prevent moisture uptake.

Mid-infrared spectra of green and roasted coffee samples processed by W, SD and Dr methods were acquired using an FTIR spectrophotometer (Cary 630, Agilent Technologies, USA) equipped with a horizontal attenuated total reflectance accessory containing a diamond crystal and ZnSe optical interface (Fig. 13). Approximately 1 g of ground coffee sample was placed onto the ATR crystal and compressed to ensure adequate contact and reproducible spectral acquisition. Spectra were collected in the range of 4000-650 cm^–1^ with a spectral resolution of 2 cm^–1^, using 16 scans per measurement and automatic background correction. Each spectrum consisted of approximately 1798 spectral variables. All measurements were performed under controlled dry atmospheric conditions at room temperature (20 ± 0.5°C) to minimize environmental interference and ensure spectral reproducibility.

Prior to PLS-DA modeling, the raw spectra were subjected to different preprocessing strategies, including baseline correction, SNV, MSC, D1 and D2 (see DATA DESCRIPTION section). These preprocessing methods were applied to reduce baseline variability, minimize scattering effects, normalize spectral intensity differences and enhance subtle spectral features associated with the chemical fingerprint of the coffee samples [11,31].

In addition to full-spectrum modeling (based considering the mid-infrared spectra), MATLAB-based feature extraction scripts were developed to extract relevant parameters computed from mid-infrared spectral information. A signal-based statistical feature extraction approach was applied to the raw and preprocessed Fourier transform mid-infrared spectra of green and roasted specialty coffee samples processed by W, SD and Dr post-harvest methods. In this approach, each spectrum was treated as a one-dimensional absorbance signal distributed across the mid-infrared wavenumber range. Six spectral descriptors were computed from each individual spectrum to capture complementary information related to the magnitude, variability, distribution shape and complexity of the spectral profile. These parameters included: INT (Eq. 1), ENG (Eq.2), STD (Eq. 3), SKW (Eq. 4), KURT (Eq. 5), and ENT (Eq. 6). INT and ENG were used as parameters which related spectral magnitude [20], whereas STD quantified the dispersion of absorbance values within the spectrum. SKW described the asymmetry of the spectral intensity distribution, while KURT provided information about the peakedness and tail behavior of the spectral profile. Finally, ENT was used as a descriptor of spectral complexity or disorder, reflecting the distribution of information contained in the spectrum [32]. These features were calculated MATLAB (MATLAB® R2023a, The MathWorks Inc., Natick, MA, USA) in which “trapz”, “std”, “skewness”, “kurtosis” and “entropy” MATLAB functions were used.

(1) $$\text{INT}=\sum_{i=1}^{N}Xz_{t_{i}}wv_{i}$$

(2) $$\text{ENG}=∥X_{t}{∥}^{2}$$

(3) $$\text{ST}D_{\;}=\sqrt{\frac{\sum_{i=1}^{N}{\left(X_{t_{i}}-\bar{X_{t}}\right)}^{2}}{\left(N-1\right)}}$$

(4) $$\text{SKW}=\frac{\left[\sum_{i=1}^{N}{\left(X_{t_{i}}-\bar{X_{t}}\right)}^{3}\right]/\left(N-1\right)}{{\sigma_{t}}^{3}}$$

(5) $$\text{KURT}=\frac{\left[\sum_{i=1}^{N}{\left(X_{t_{i}}-\bar{X_{t}}\right)}^{4}\right]/\left(N-1\right)}{{\sigma_{t}}^{4}}$$

(6) $$\text{ENT}=-\sum_{i=1}^{N}p\left(X_{t_{i}}\right)\text{lo}g_{2}p\left(X_{t_{i}}\right)$$

Where Xt is the mid-infrared spectrum, Xzt corresponded with the positive values of Xt, X̅t is the mean of each mid-infrared spectrum, N is the number of elements of each spectrum, wv is the vector which registered the wavenumbers (cm^–1^), σt the standard deviation of each mid-infrared spectrum and p(Xti) is the probability of the occurrence of the i-*th* absorbance value in the discretized mid-infrared spectrum.

The R-based computational pipeline was developed using the statistical software R (R Core Team, 2025), to calibrate and validate PLS-DA models for the multivariate classification of specialty coffee samples processed by W, SD and Dr. The script allows the user to interactively select the spectral preprocessing dataset, define the modeling approach as either full-spectrum or feature-extracted modeling, and specify the coffee stage to be analyzed as green coffee, roasted coffee or both stages simultaneously. The pipeline also performs the conversion of dummy-coded post-harvest classes into categorical responses, stratified training and validation partitioning, model calibration, sample prediction, performance assessment through confusion matrices and classification metrics, and graphical visualization of scores, loadings, variable importance and diagnostic plots. This structure enables the systematic assessment of model performance under different combinations of spectral preprocessing, data representation and coffee stage.

## Limitations

This work represents an initial step toward the development of intelligent systems for coffee classification, authentication, quality assessment, and fraud detection based on spectroscopic information and multivariate modeling. The proposed dataset and analytical framework demonstrate the potential of mid-infrared spectroscopy combined with PLS-DA to describe discriminant spectral patterns associated with coffee post-harvest processing. Nevertheless, despite including 162 samples, the current dataset may still impose some limitations on the assessment of model robustness and generalizability, particularly when extending the models to broader and more heterogeneous coffee populations. Although the proposed modeling framework incorporates internal cross-validation and independent holdout validation, future studies should substantially increase the number of samples to enable more reliable model calibration, validation, and assessment of performance stability. In addition to increasing sample size, future datasets should encompass greater biological, geographical, and technological variability by including different coffee cultivars, geographical origins, growing environments, harvest seasons, production batches, post-harvest processing conditions, drying protocols, storage conditions, and roasting treatments. The incorporation of such heterogeneous and independently collected samples would provide a more rigorous basis for evaluating model robustness and transferability. More comprehensive datasets could also support the development of models capable of distinguishing not only post-harvest processing methods, but also geographical origin, cultivar, quality attributes, atypical samples, and potentially adulterated or defective batches. At the industrial level, these tools could facilitate rapid, objective, and non-destructive screening of coffee lots and support data-driven decision-making throughout processing, storage, commercialization, and quality-control operations.

## Ethics Statement

The dataset acquired in this study did not involve human subjects, animal experiments, or data obtained from social media platforms.

## CRediT Author Statement

**Gentil A. Collazos-Escobar:** Conceptualization, Methodology, Software, Data curation, Visualization, Writing-original draft, Writing-review and editing. **Andrés F. Bahamon-Monje:** Software, Data curation, Writing-original draft, Writing-review and editing. **Nelson Gutiérrez-Guzmán:** Conceptualization, Funding acquisition, Project administration, Resources, Supervision, Validation, Writing-review, and editing.

## Data Availability

Mendeley Datahttps://data.mendeley.com/datasets/9x3ns2f6vr/2.
